# Processing methods and mechanisms for saponin-rich traditional Chinese medicines: a review

**DOI:** 10.1186/s13020-025-01264-1

**Published:** 2025-12-02

**Authors:** Shu-qing Tian, Xiao-ting Wang, Ya-zhu Wang, Jia-qi Shi, Hui Gao

**Affiliations:** 1 School of Pharmacy, Liaoning University of Traditional Chinese Medicine, Dalian, 116600 Liaoning China; 2 Traditional Chinese Medicine Processing Technology Inheritance Base (Liaoning) of the National Administration of Traditional Chinese Medicine, Dalian, 116600 China; 3 Liaoning Provincial Traditional Chinese Medicine Processing Technology Innovation Center, Dalian, 116600 China

**Keywords:** Saponins, Saponin-rich traditional Chinese medicines, Chinese medicine processing

## Abstract

As an important class of bioactive components in TCM, saponins exhibit various significant pharmacological effects. Therefore, investigating the impacts of processing on the chemical components and action mechanisms of saponin-rich TCMs is of great importance. This review aims to summarize the effects of different processing methods on saponin components in TCMs and the chemical transformations of saponins during processing, while also prospecting future research directions. A bibliographic investigation was performed by examining the available data on saponin-rich TCMs from globally accepted scientific databases and search engines (Pubmed, Scopus and Web of Science, SciFinder and Google Scholar). Different processing methods cause distinct saponin changes, significantly affecting their physicochemical properties and pharmacological effects. Understanding these effects and transformations is crucial for developing targeted saponin preparations, aiding clinical use of such TCMs and mechanism research.

**Graphical Abstract:**

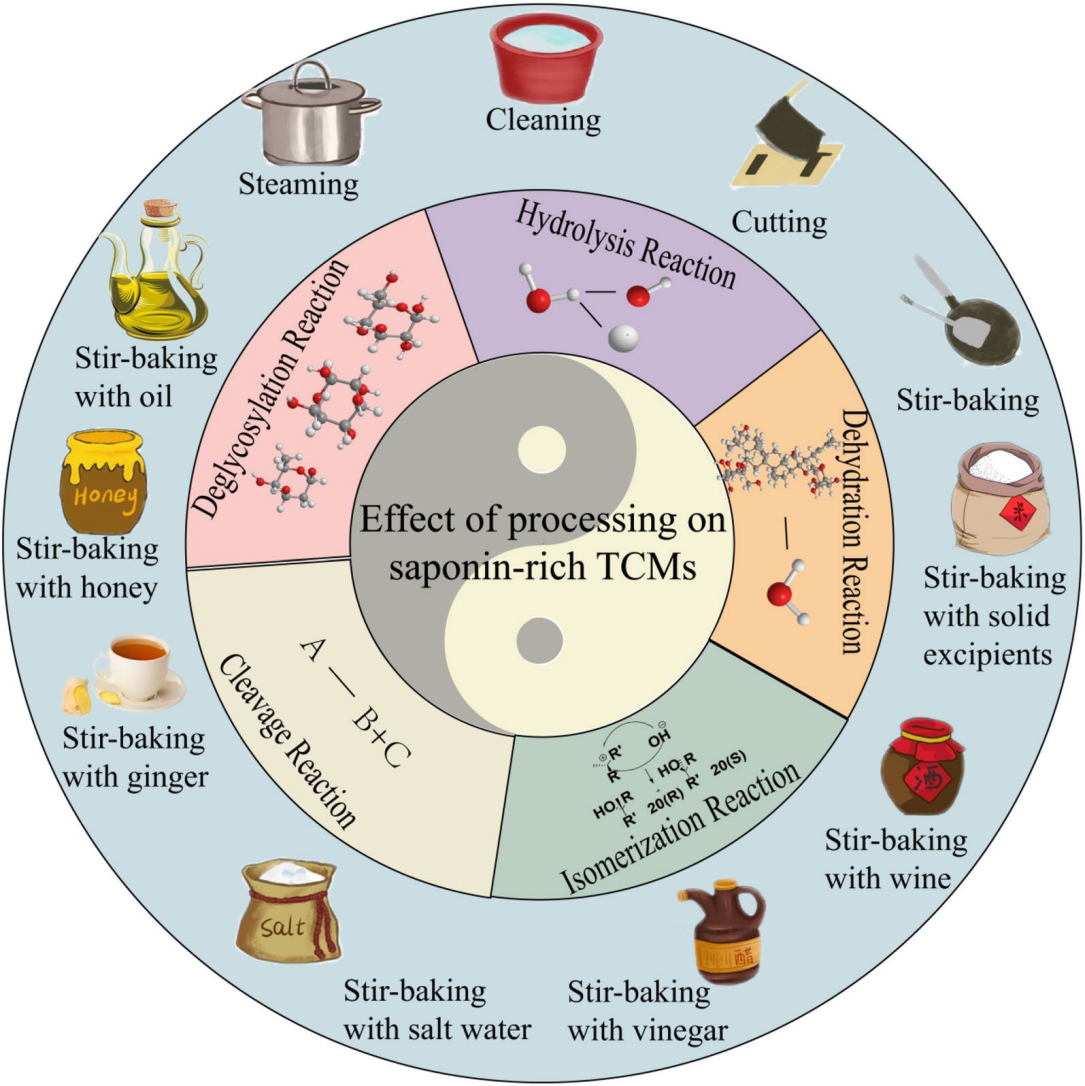

## Introduction

Traditional Chinese medicines are primarily derived from natural sources such as plants, animals, and minerals. However, these crude medicinal materials are generally unsuitable for direct clinical application and must be processed into decoction pieces before being used in clinical practice. The processing of Chinese herbal medicines is a pharmaceutical technology that processes crude drugs according to the Traditional Chinese Medicine theory and individual crude drugs nature, and the requirements of drug dispensing, pharmaceutical preparation, and clinical use. Generally, the processing methods of TCM mainly include cleaning, cutting, simple stir-baking, stir-baking with solid excipients, stir-baking with liquid excipients, steaming, boiling, etc. Processing of Chinese herbal medicines, as an ancient and unique traditional technique, can enhance therapeutic efficacy, reduce or eliminate drug toxicity and side effects, or modify medicinal properties and therapeutic directions, while ensuring medication safety and clinical effectiveness. Numerous modern studies have shown that after the processing of traditional Chinese medicines, the alterations in their pharmacological effects or toxicity are due to factors such as heating and the addition of water during the processing. These factors lead to the changes in many chemical components, including saccharides, saponins, alkaloids, and flavonoids, etc. While there exist comprehensive reviews on compositional changes in alkaloid-rich Chinese herbal medicines after processing, a systematic review focusing specifically on saponin-rich herbs remains lacking. Saponins represent a crucial class of bioactive constituents in TCM, exhibiting diverse pharmacological effects [1] including antimicrobial [2], antipyretic [3], sedative [4], anticancer, antitumor [5], and hypoglycemic activities. Numerous saponin-rich Chinese herbal medicines undergo complex structural transformations of their saponin constituents during processing, achieving the dual pharmaceutical objectives of toxicity reduction and efficacy enhancement. As exemplified by *Panax ginseng*, revered as the ‘King of Medicinal Herbs’, it boasts a millennia-long history of therapeutic use in China. Recognized for its ability to tonify primordial qi, restore pulse and arrest collapse, generate bodily fluids, and tranquilize the mind, after being steamed to make red ginseng, it has the effects of greatly replenishing primordial qi, restoring pulse and consolidating collapse, and invigorating qi to control bleeding. Post-processing, its anticancer efficacy and hydroxyl radical scavenging capacity are significantly amplified. Scientific studies attribute these modifications to deglycosylation [6] and isomerization [7, 8] of saponin constituents, demonstrating that structural transformations in saponins directly drive the evolution of ginseng’s pharmacological actions. The toxic herb *Phytolacca acinosa* has been processed with vinegar for over a millennium in China to attenuate its toxicity. Its primary toxic constituents-esculentosides B (EsB), C (EsC), and H (EsH) are triterpenoid saponins. After processing, the toxicity is significantly attenuated. Analytical results demonstrate that the contents of EsB, EsC and EsH decrease due to hydrolysis reactions during processing. This structural transformation of saponin components directly accounts for the toxicity reduction of *Phytolacca acinosa*. In light of this, in-depth and sustained investigation into the effects of processing methods on saponin constituents in TCMs holds significant implications for Chinese herbal medicines processing research.

In recent years, the field of TCM processing has witnessed rapid development, with a series of related research studies on herbal processing techniques being successively conducted. Numerous studies have documented the effects of processing methods on saponin-containing traditional Chinese medicinal herbs. This paper presents a systematic review on the effects of different processing methods on saponin constituents in TCMs and the chemical transformations of saponins during processing. Through comprehensive organization and integrative analysis, we aim to establish a thorough and in-depth understanding of these processes, thereby expecting to provide a comprehensive and reliable reference for the rational processing and further development of saponin-rich Chinese medicines.

## Impact of processing methods on saponin-rich traditional Chinese medicines

Processing methods hold great significance for traditional Chinese Medicines rich in saponins. Various processing methods can alter the appearance, texture, and solubility of medicinal materials through physical effects. Meanwhile, certain chemical reactions occur during the processing, which prompt changes in saponin components. As a result, these have a profound impact on the content, structural stability, and biological activity of saponins. As shown in Table 1.

**Table 1:** Saponin-rich traditional Chinese medicines and saponin changes during processing

Processing methods		Chinese materia medica	Original	Medicinal parts	Changes in saponins	Changes in pharmacological effects	Processing purpose	Saponin reaction types	References
Cleaning		*Radix Bupleuri*	*Bupleurum chinense* DC.; *Bupleurumscorzonerifolium*Willd	Radix	Total saikosaponins, saikosaponin A, D (↑)	Same as thecrude medicinal material	Remove impurities and non-medicinal parts to ensure the purity of the drug		[13]
		*Polygalae Radix*	*Polygala tenuifolia* Willd.; *Polygala sibirica* L	Cortex Radicis	Total saponins of Polygala (↑)	Same as thecrude medicinal material			[15]
		*Panax ginseng *(Ginseng)	*Panax ginseng* C.A.Mey	Radix	Total ginsenosides, ginsenoside Rg5, Rk1, Rg1, Re, Rg3, Rh1 (↑)	Same as thecrude medicinal material			[132]
		*Panax notoginseng *(Notoginseng)	*Panax notoginseng *(Burk.) F.H.Chen	Radix	ginsenoside Rg1,Re,Rb1,Rd, notoginsenoside R1 (↑)	Same as thecrude medicinal material			[133]
		*Paris polyphylla*	*Paris polyphyllaSmith* var.chinenisi (Franch) Hara	Rhizoma	Steroidal saponins, paris saponin H, paris saponin VI (↑)	Same as thecrude medicinal material			[134]
		*Panax quinquefolius *(American ginseng)	*Panax quinquefolius *L	Radix	American ginseng saponins (↑)	Same as thecrude medicinal material			[135]
		*Platycodon grandiflorus*	*Platycodon grandiflorum *(Jacq.) A.DC	Radix	Total platycodin saponins (↑)	Same as thecrude medicinal material			[136]
		*Tribulus terrestris*	*Tribulus terrestris* L	Fructus	Diosgenin (↑)	Same as thecrude medicinal material			[137]
Cutting		*Panax quinquefolius *(American ginseng)	*Panax quinquefolium* L	Radix	Total saponins, ginsenoside Rb1, Rc, Re, Rd, Ro (↑)	Same as thecrude medicinal material	Facilitate the extraction of active ingredients, subsequent processing, prescription and preparation, and storage		[138]
		*Gynostemma pentaphyllum*	*Gynostemma pentaphyllum* (Thumb.) Makino	Herba	Rare saponins of Gynostemma pentaphyllum (↑)	Same as thecrude medicinal material			[139]
		*Glycyrrhiza uralensis*(licorice)	*Glycyrrhiza uralensis*Fisch.; *Glycyrrhiza inflata*Bat.; *Glycyrrhiza glabra*L	Radix	Total glycyrrhizic saponins (↑)	Same as thecrude medicinal material		Hydrolysis Reaction	[140]
		*Panax ginseng *(Ginseng)	*Panax ginseng* C.A.Mey	Radix	Ginsenoside Rg1, Re, Rb1, Ro(↑)	Same as thecrude medicinal material		Hydrolysis Reaction	[141]
		*Stellaria Dichotoma*	*Stellaria dichotoma* L.var. lanceolata Bge	Radix	Total saponins (↑)	Same as thecrude medicinal material			
Simple stir-baking		*Ziziphi SpinosaeSemen*	*Ziziphus jujuba* Mill.var.spinosa (Bunge) Hu ex H.F.Chou	Semen	Jujuboside A, B (↑)	Sleep-promoting effect (↑)	Enhance therapeutic efficacy		[142]
		*Tribulus terrestris*	*Tribulus terrestris* L	Fructus	tribulus sapogenins, tribuluside A, furostanol saponin B and tribuloside K, hecogenin and tigogenin (↑); Cmax of tribuluside A, furostanol saponin B and tribuloside K (↑); Total tribulus saponins, terrestrosin D and 25R-tribulosin (↓);	Therapeutic effects on hepatitis, inflammation, and cardiovascular diseases (↑)		Deglycosylation Reaction	[28, 143]
		*Glycyrrhiza uralensis *(licorice)	*Glycyrrhiza uralensis *Fisch.; *Glycyrrhiza inflata*Bat.; *Glycyrrhiza glabra*L.	Radix	Glycyrrhizic acid (↑)	Immunomodulatory effect (↑)		Hydrolysis Reaction	[145, 146]
		*Anemarrhenae Rhizoma*	*Anemarrhena asphodeloides*Bge	Rhizoma	timosaponin BⅡ, sarsasapogenin、timosaponinⅠ(↑)	Yin-nourishing effect (↑) Antioxidant activity (↑)			[147, 148]
Stir-baking with solid excipients		*Panax ginseng *(Ginseng)	*Panax ginseng* C.A.Mey	Radix	20(S)-Rg2, 20(S)-Rh1, 20(R)-Rh1, F2, 20(S)-Rg3, 20(R)-Rg3, 20(S)-Rs3, 20(R)-Rs3(↑)	Same as thecrude medicinal material	Enhance therapeutic efficacy	Deglycosylation Reaction	[36]
Stir-baking with liquid excipients	Stir-baking with wine	*Radix Bupleuri*	*Bupleurum chinense*DC.; *Bupleurumscorzonerifolium*Willd	Radix	total saponins, saikosaponin b1, saikosaponin b2 (↑); saikosaponin a, saikosaponin d (↓)	Blood-activating and stasis-resolving effect (↑); anti-inflammatory effect (↓); antidepressant effect (↑)	1. conduct the drug to distributing upward 2. Enhance therapeutic efficacy	HydrolysisReaction	[149, 150]
		*Dipsaci Radix*	*Dipsacus asper* Wall.Ex Henr	Radix	Dipsacoside B, dipsacoside VI, three acetylated analogues of dipsacoside VI (↑)	Analgesic, anti-inflammatory, anticoagulant effects, and prevention of recurrent spontaneous abortion (RSA) (↑)		Hydrolysis Reaction	[151, 152]
		*Clematis chinensis*	*Clematis manshurica* Rupr.; *C.hexapetala* Pall.; *chinensis Osbeck*	Radix	Hederagenin (↑)	Anti-inflammatory and analgesic effects (↑)			[153, 154]
		*Achyranthes bidentata*	*Achyranthes bidentata* Bl	Radix	Ginsenoside Ro (↑); zingibroside R1, achyranthoside Ⅰ and chikusetsusaponin Ⅳ (↓)	Blood-activating and stasis-resolving effect (↑)		Deglycosylation Reaction	[155, 156]
		*Anemarrhenae Rhizoma*	Anemarrhena asphodeloides Bge	Rhizoma	timosaponin Ⅰ、timosaponin AⅢ(↑)	Hypoglycemic and hypolipidemic effects (↑)		Deglycosylation Reaction	[147]
	Stir-baking with vinegar	*Radix Bupleuri*	*Bupleurum chinense*DC.; *Bupleurumscorzonerifolium*Willd	Radix	Saikosaponin b1, saikosaponin b2, saikosaponin g, saikosaponin h, saikosaponin i, saikosaponin f, acetylsaikosaponin a, acetylsaikosaponin b2, diacetylsaikosaponin a, diacetylsaikosaponin b2 (↑)	Antifibrotic effect (↑)	1. Guide the drug into the liver 2. Enhance therapeutic efficacy	Hydrolysis Reaction、Dehydration Reaction	[157, 158]
		*Sparganii Rhizoma*	*Sparganium stoloniferum* Buch.-Hma	Tuber	Total sparganium saponins (↑)	Therapeutic effect on hyperlipidemia (↑)			[159]
		*Phytolaccae Radix*	*Phytolacca acinosa*.; *americana*	Radix	Total phytolacca saponins; phytolaccoside A (↑); phytolaccoside H, phytolaccoside B, phytolaccoside C (↓)	Hepatorenal toxicity (↓); diuretic-promoting effect (↑)		Hydrolysis Reaction	[160]
		*Achyranthes bidentata*	*Achyranthes bidentata* Bl	Radix	Ginsenoside Ro, chikusetsusaponin Ⅳa(↑)	Same as thecrude medicinal material			[161]
	Stir-baking with salt water	*Anemarrhenae Rhizoma*	*Anemarrhena asphodeloides *Bge	Radix	Smilagenin, timosaponin AIII, timosaponin BIII(↑); Cmaxtimosaponin AIII, timosaponin BIII(↑) timosaponin E(↓)	Diabetic cognitive impairment (↑) Laxative and bowel-regulating effect (↑)Hypoglycemic effect (↑)	1. Guide the drug into the kidney; 2. Enhance therapeutic efficacy; 3. Alleviate pungent and dry properties	Deglycosylation Reaction、Cleavage Reaction	[64, 69, 70, ]
									[162, 163]
		*Dipsaci Radix*	*Dipsaci asper*Wall.ex Henry	Radix	Dipsacoside Ⅵ (↑)	Kidney-Yang deficiency (↑)			[164, 165]
		*Trigonella foenum-graecum*	*Trigonella foenum-graecum*L	Semen	Diosgenin, spirostanol-type/isospirostanol-type steroidal saponins (↑); furostanol-type steroidal saponins (↓)	Hypolipidemic effect (↑)			[84, 165, 166, ]
		*Achyranthes bidentata*	*Achyranthes bidentata* Bl	Radix	Cmax of ginsenoside Ro and chikusetsusaponin IVa (↑); AUC0-t of ginsenoside Ro and chikusetsusaponin IVa (↑)	Anti-osteoarthritis effect (↑); renal nourishing and protective effect (↑)			[167, 168]
	Stir-baking with honey	*Lilium*L	*Lilium lancifolium*Thunb.; *Lilium brownii*F.E.Brownvar.viridulumBaker.; *Lilium pumilum*DC	Bulbus	Total lily saponins (↑)	Same as thecrude medicinal material	1. Enhance therapeutic efficacy; 2. Moderate the property of the drug		[169]
		*Astragali Radix*	*Astragalus membranaceus *(fisch.) Bge.var.mongholicus (Bge.) Hsiao; *A. membranaceus* (Fisch.) Bge	Radix	Astragaloside IV, astragaloside I, total astragalus saponins (↑); astragaloside II, astragaloside I (↓)	Qi-tonifying effect (↑)			[170, 171]
		*Polygalae Radix*	*Polygala tenuifolia* Willd.;*Polygala sibirica* L	Radix	tenuifolin (↑); Total polygala saponins; polygala sapogenins (↓);	Expectorant and antitussive effects (↑); toxicity (↓)		Cleavage Reaction、Hydrolysis Reaction	[107, 111]
		*Glycyrrhiza uralensis*(licorice)	*Glycyrrhiza uralensis*Fisch.; *Glycyrrhiza inflata*Bat.; *Glycyrrhiza glabra*L	Radix	Cmax of glycyrrhetinic acid (↑); Glycyrrhizic acid (↓)	Cardiotoxicity (↓) Immunomodulatory activity (↑)		Hydrolysis Reaction	[172]
									[135]
	Stir-baking with ginger juice	*Platycodon grandiflorus*	*Platycodon grandiflorum *(Jacq.)A.DC	Radix	Platycodin D (↑)	Antitussive and expectorant effects (↑)	1. Enhance therapeutic efficacy; 2. Alleviate side effects		[80]
		*Panax quinquefolius *(American ginseng)	*Panax quinquefolium* L	Radix	Ginsenoside Re, ginsenoside Rb2 (↑) GinsenosideRh7, GinsenosideRh8, GinsenosideRh10, GinsenosideC-K(↑)	Acute kidney injury (↑)		Deglycosylation Reaction	[173]
									[86]
	Stir-baking with oil	*Panax notoginseng *(Notoginseng)	*Panax notoginseng *(Burk.) F.H.Chen	Radix	ginsenoside Rg6, Rg4, Rk3, Rh4, Rk1, Rg5, Rh1 (↑); notoginsenoside R1, ginsenoside Rg1, Re, Rb1 (↓)	Same as thecrude medicinal material	1. Enhance therapeutic efficacy; 2. Facilitate crushing	Deglycosylation Reaction	[113]
Steaming		*Polygonati Rhizoma*	*Polygonatum kingianum* Collett & Hemsl	Radix	diosgenin, total saponins (↑); Protodioscin, narcissoside, dioscin (↓)	Toxicity (↓); therapeutic effect on kidney injury (↑), antioxidant effect (↑), anti-inflammatory effect (↑)	1. Preserve medicinal effect and facilitate storage; 2. Facilitate softening and slicing; 3. Enhance therapeutic efficacy	Deglycosylation Reaction、Isomerization Reaction	[174, 175]
		*Bolbostemma paniculatum*	*Bolbostemma paniculatum *(Maxim) Franquet	Tuber	Total bolbostemma saponins (↑)	Antitumor effect (↑)			[176]
		*Panax ginseng *(Ginseng)	*Panax ginseng* C.A.Mey	Radix	Ginsenoside 20 (S)-Rg3, 20 (R)-Rg3, 20 (S)-Rh1, 20 (R)-Rh1, 20 (R)-Rg2 (↑)	Immunomodulatory, anti-inflammatory, and antiproliferative effects (↑)		Hydrolysis Reaction、Dehydration Reaction、Isomerization Reaction	[177]
		*Panax quinquefolius *(American ginseng)	*Polygala tenuifolia* Willd.;*Polygala sibirica* L	Radix	Rb2、Rb3、20(S) -Rg3,Rb1、Rb2、Rb3、20(S) -Rg3(↑)	Therapeutic effect on myocardial injury (↑); antioxidant activity and antiproliferative activity against colon cancer cells (↑)		Hydrolysis Reaction、Dehydration Reaction、Isomerization Reaction	[127, 173]
		*Codonopsis pilosula*	*Codonopsis pilosula* (Franch.)Nannf	Radix	Total saponins (↑)	Therapeutic effect on skin inflammation (↑); immunological activity (↑)			[178, 179]
		*Panax notoginseng *(Notoginseng)	*Panax notoginseng *(Burk.) F.H.Chen	Radix	Notoginsenoside R1, ginsenoside Rg1, Re, Rb1 (↓); 20 (S)-ginsenoside Rg3 and 20 (R)-ginsenoside Rg3(*)	Immunomodulation (↑); antioxidant effect (↑)		Hydrolysis Reaction、Dehydration Reaction、Isomerization Reaction	[180]

↑, increased; ↓, decreased; *, new produced

### Cleaning

Derived from nature, TCMs are often contaminated with sand, mud, and foreign objects during collection, processing, transportation, and storage, and are prone to insect infestation and mildew damage, which must be removed. TCMs vary in their medicinal parts—some use roots, some flowers, some barks, and so on—and non-medicinal parts need to be removed. The removal of impurities and non-medicinal parts is referred to as cleaning processing.

For example, *Radix Bupleuri*, the root of *Bupleurum chinense DC.*, has various remarkable effects such as anti-inflammatory and analgesic [8], anti-depressive [9], anti-tumor [10], and anti-fibrotic effects [11]. *Radix Bupleuri* contains a large amount of saikosaponins, which are the active ingredients responsible for the above-mentioned effects, with the content in the root being significantly higher than that in the stem, leaf, and flower and other parts [12, 13]. Therefore, there is a scientific basis for using the roots of *Bupleurum chinense DC.* as medicine and not using the stems, leaves, and flowers since ancient times. The research on chemical components not only verifies the correctness of the ancient people’s medication habits but also further promotes the development of the cleaning methods of traditional Chinese medicines. For example, *Polygalae Radix* has pharmacological effects such as neuroprotection, improvement of memory function, anti-depression, anti-insomnia, and anti-anxiety, with saponins being the main active ingredients. In ancient times, *Polygalae Radix* was used without its pith, yet modern research has found that the chemical components in the roots, root barks, and pith are similar. Although the root bark has the highest saponin content, followed by the root and then the pith, studies on acute toxicity, hemolysis, sedation, anticonvulsant, antitussive and expectorant effects have shown that using *Polygalae Radix* without removing the pith does not significantly affect its key pharmacological effects and safety [14–16]. Moreover, the 2025 edition of the Chinese Pharmacopoeia allows the use of *Polygalae Radix* without removing the pith. These facts fully demonstrate that traditional medication methods are continuously adjusted and improved with the deepening of scientific research and the accumulation of practical experience.

**Fig.1:**
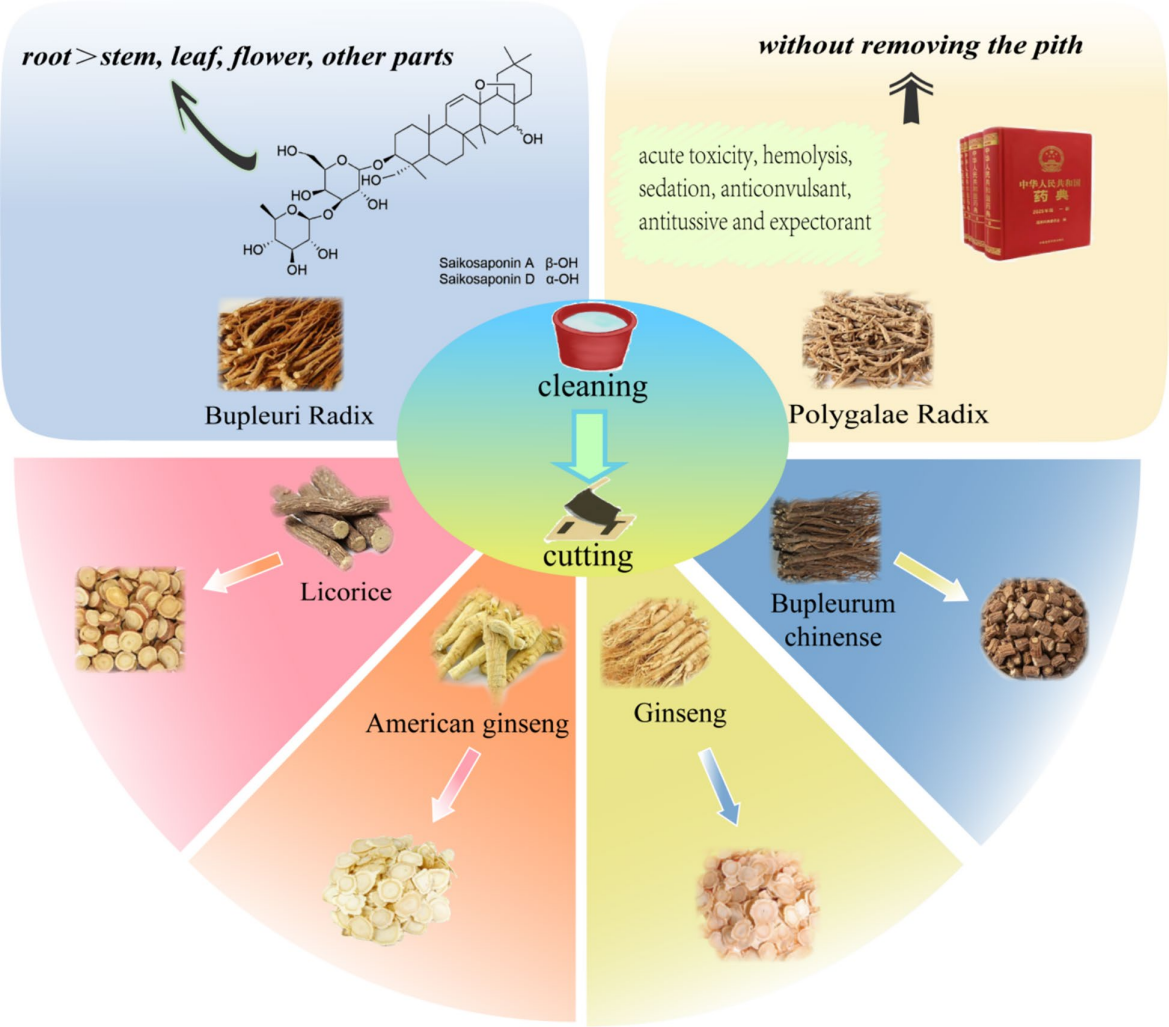
Effects of cleaning and cutting on saponin-rich TCMs

Overall, cleaning influences saponin-rich TCMs by removing impurities and non-medicinal parts. The above findings not only verifies the scientific validity of the ancient medication habits to a certain extent, but also provides a scientific basis for the change and development of modern medication methods, which contributes to the continuous optimization of traditional Chinese medicine processing in clinical applications(Fig. 1).

### Cutting

For dry and hard medicinal materials, they must first be softened to an appropriate hardness, then cut into pieces of suitable size, and subsequently dried; this constitutes the traditional cutting method for Chinese medicinal materials. After being processed by this traditional cutting method, TCMs are not only facilitates further processing, formulation, identification, and storage, but also have their shapes changed, surface areas increased, and the dissolution of chemical components promoted [17]. In recent years, there have been many studies on cutting methods, which have carried out reforms in aspects such as the softening method before cutting, the cutting method, and the drying method after cutting. These studies involve saponin-rich TCMs, including *Glycyrrhiza uralensis*, *Panax quinquefolius*, *Panax ginseng*, *Radix Bupleuri*, etc.

For example, the traditional softening method for *Glycyrrhiza uralensis* (Licorice) is moistening by spraying water. It is worth noting that the active ingredient in licorice is glycyrrhizic acid, an oleanane-type saponin that is readily soluble in water and prone to hydrolysis reaction which causes loss during the sprinkling and moistening softening process; for details of the process, sect 3.1 “Hydrolysis Reaction” below [18]. Therefore, some researchers reformed the softening method of licorice using the total saponin content as an index, and found that steaming and moistening could reduce the loss of saponins in water and increase the total saponin content in licorice. Hence, compared with the traditional method of moistening by spraying water, the steaming and moistening method is more suitable for the softening and processing of licorice, for the reason that it avoids the excessive moisture in the traditional water-spraying moistening process which causes saponins to undergo hydrolysis reactions. For *Panax quinquefolius* (American ginseng), natural drying is the traditional drying method, during which its active components, ginsenosides Rg1, Re, and Rb1, are prone to loss. Therefore, some researchers have studied its drying methods and found that air impingement drying at 45 °C can significantly reduce the loss of ginsenosides Rg1, Re, and Rb1 [19, 20], providing a practical direction for the innovation of American ginseng’s drying technology. The fresh-cut processing has emerged as a technology garnering increasing attention and widespread application in the field of TCMs processing in recent years, which can circumvent issues such as active ingredient loss caused by prolonged processing time and repeated production steps during cutting, as well as obstacles to component dissolution—including cellular shrinkage and cell wall thickening induced by drying—which hinder the release of chemical constituents. This method better preserves saponins within cells and facilitates their dissolution during subsequent decoction and other processes. Numerous saponin-rich TCMs, including *Panax ginseng* (Ginseng) [21, 22] and *Radix Bupleuri *[23], have been successfully processed using this technique.

Therefore, appropriate softening, cutting, and drying methods can promote the dissolution of saponins in saponin-rich TCMs and reduce the loss of saponins. In actual production, the most suitable cutting method should be selected based on the characteristics of medicinal materials to significantly improve the quality of saponin-rich TCMs (Fig. 1).

This figure presents two fundamental processing steps: cleaning and cutting. The cleaning process focuses on *Bupleuri Radix* and *Polygalae Radix*, achieving spatial enrichment of saponins by removing impurities and non-medicinal parts. The cutting process centers on *Glycyrrhizae Radix et Rhizoma*, *Panacis Quinquefolii Radix*, and *Ginseng Radix et Rhizoma*, improving saponin utilization efficiency through optimized cutting techniques such as softening and drying. It clearly demonstrates the progressive role of these two steps in optimizing the quality of saponin-rich TCMs, with a core focus on two objectives: “removing impurities” and “retaining/promoting saponin dissolution”.

### Stir-baking

Stir-baking refers to the processing method in which the cleansed or cut drugs are stirred and rotated, with or without excipients added, in a preheated frying container, and in which different fire levels are continuously applied until the drugs reach a desired degree of processing. According to the stir-frying operation and whether excipients are added, it can be classified into three categories: simple stir-baking, stir-baking with solid excipients, and stir-baking with liquid excipients.

#### Simple stir-baking

**Fig.2:**
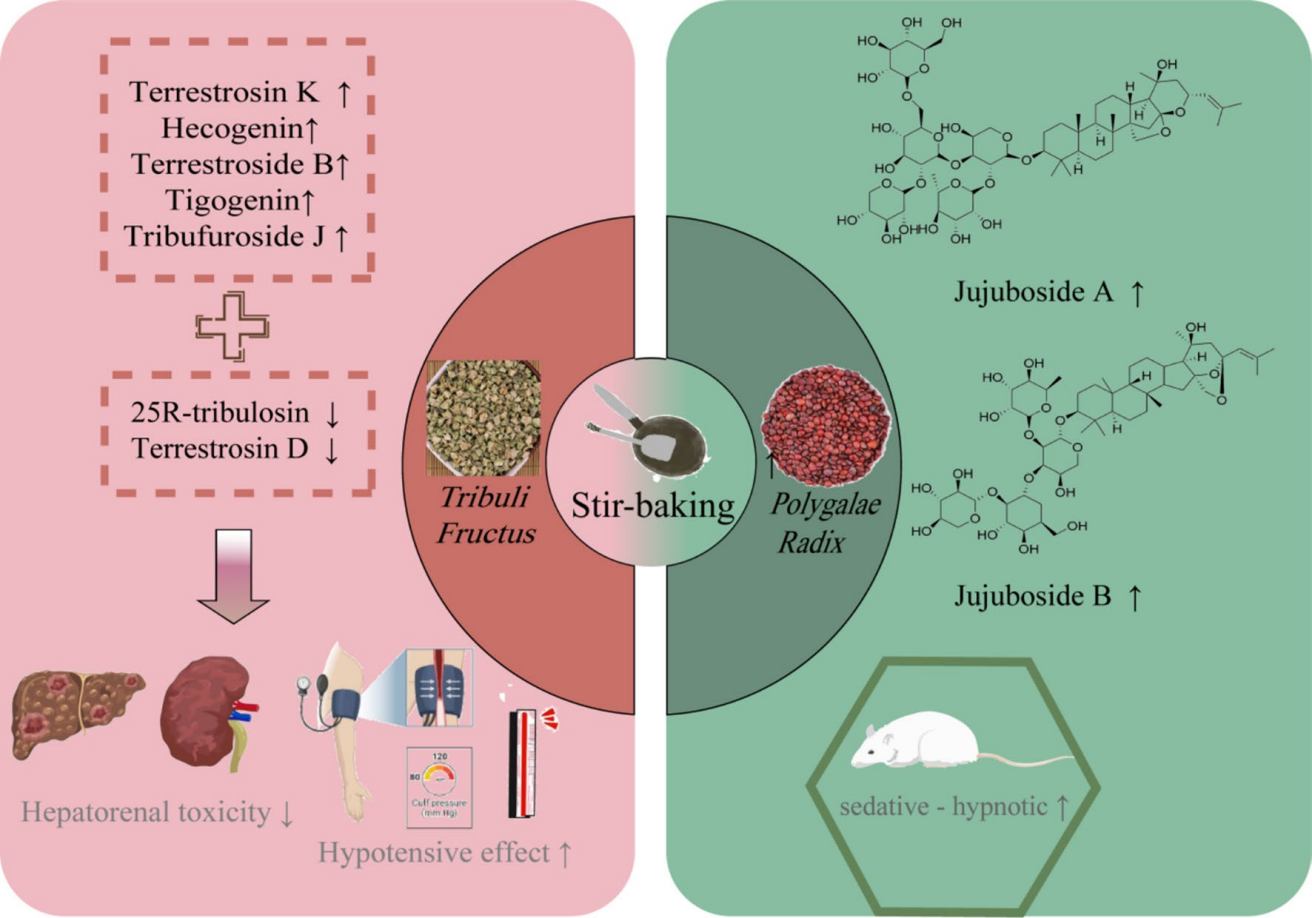
Effects of simple stir-baking on saponin-rich TCMs

Stir-baking without any excipients is called Simple stir-baking, which has been widely used since the Han Dynasty and remains one of the oldest and most fundamental processing methods in TCMs (Fig. 2). *Ziziphi Spinosae Semen* is a representative medicinal material processed by simple stir-baking, which exhibits functions of nourishing the liver, calming the mind, arresting sweating, and promoting salivation. Its main components include damarane-type triterpenoid saponins, such as jujuboside A and B, which serve as the key pharmacodynamic material basis for tranquilizing and sedative-hypnotic effects [4]. After simple stir-baking, the contents of jujuboside A and B in *Ziziphi Spinosae Semen* increase [24], enhancing its sedative-hypnotic effects. *Tribulus terrestris* exhibits the effects of calming the liver and relieving depression, as well as activating blood and benefiting qi. Its primary active components are saponin compounds, which are used in the treatment of hypertension, cardiovascular diseases, and sexual dysfunction [25]. Among them, Terrestrosin K, Hecogenin [26–30], Terrestroside B, Tigogenin, and Tribufuroside J [26] have been demonstrated to treat essential hypertension [31]. After simple stir-baking of *Tribulus terrestris*, the content of these components and the highest concentration of furosteroidal saponins (such as tribuluside A, Terrestroside B, and Terrestrosin K) in brain tissue increased [32], which enhanced the therapeutic effect on hypertension [31] and cardiovascular diseases. Simple stir-baking of *Tribulus terrestris* not only enhances its efficacy but also reduces toxicity. Hepatorenal toxicity components in *Tribulus terrestris*—Terrestrosin D and 25-R-Tribulosin—are detected to undergo deglycosylation reactions and thereby converted into steroidal sapogenins [33] with negligible hepatorenal toxicity after stir-baking, resulting in reduced hepatorenal toxicity. The reason for the reduced toxicity and enhanced efficacy of stir-baked *Tribulus terrestris* is that stir-baking promotes the dissolution and transformation of saponins, as well as increases their bioavailability.

Aking *Ziziphi Spinosae Semen* and *Tribuli Fructus *as research objects, this figure illustrates the dual effects of simple stir-baking on saponin-rich TCMs. For *Ziziphi Spinosae Semen*, simple stir-baking breaks the seed coat, increasing the content of jujuboside A/B and enhancing sedative-hypnotic efficacy. For *Tribuli Fructus*, simple stir-baking increases the content of active saponins while reducing toxic saponins, achieving efficacy enhancement and toxicity reduction. Meanwhile, it establishes the correlation between changes in saponin components and pharmacological effects.

#### Stir-baking with solid excipients

Stir-baking with solid excipients refers to a processing method in which the cleaned or cutted TCMs are heated and stir-baked together with solid excipients in a stir-baking vessel. Commonly used solid excipients include wheat bran, river sand, rice, soil, talcum powder, etc., with different excipients exerting distinct processing effects. For example, wheat bran can moderate the dryness of medicinal materials and enhance their spleen-strengthening efficacy; river sand can make hard-textured drugs crispy, facilitating the decoction of active ingredients; rice can reduce the toxicity and irritation of drugs while enhancing their spleen-strengthening and antidiarrheal effects; soil helps enhance the spleen-nourishing and antidiarrheal effects of drugs; talcum powder is often used to improve the texture of drugs and simultaneously exert heat-clearing effects, etc.

For example, Ginseng exhibits the effects of nourishing vital qi, tonifying the spleen and benefiting the lung, among others [34]. Ginseng has various processing methods, among which ginseng stir-baked with rice has its unique curative effects. Rice has sweet taste and neutral nature, fortifying spleen, harmonizing the middle, invigorating the spleen and harmonizing the stomach [35]. After stir-baking ginseng with rice, eight rare ginsenosides are produced, namely ginsenoside 20(S)-Rg2, 20(S)-Rh1, 20(R)-Rh1, F2, 20(S)-Rg3, 20(R)-Rg3, 20(S)-Rs3 and 20(R)-Rs3 [36], which can promote the body’s non-specific immunity, enhance cellular immune function, improve the body’s immune defense ability [37–41], and consequently the spleen-tonifying and stomach-nourishing effect of rice-fried ginseng is enhanced [42].

#### Stir-baking with liquid excipients

##### Stir-baking with wine

**Fig.3:**
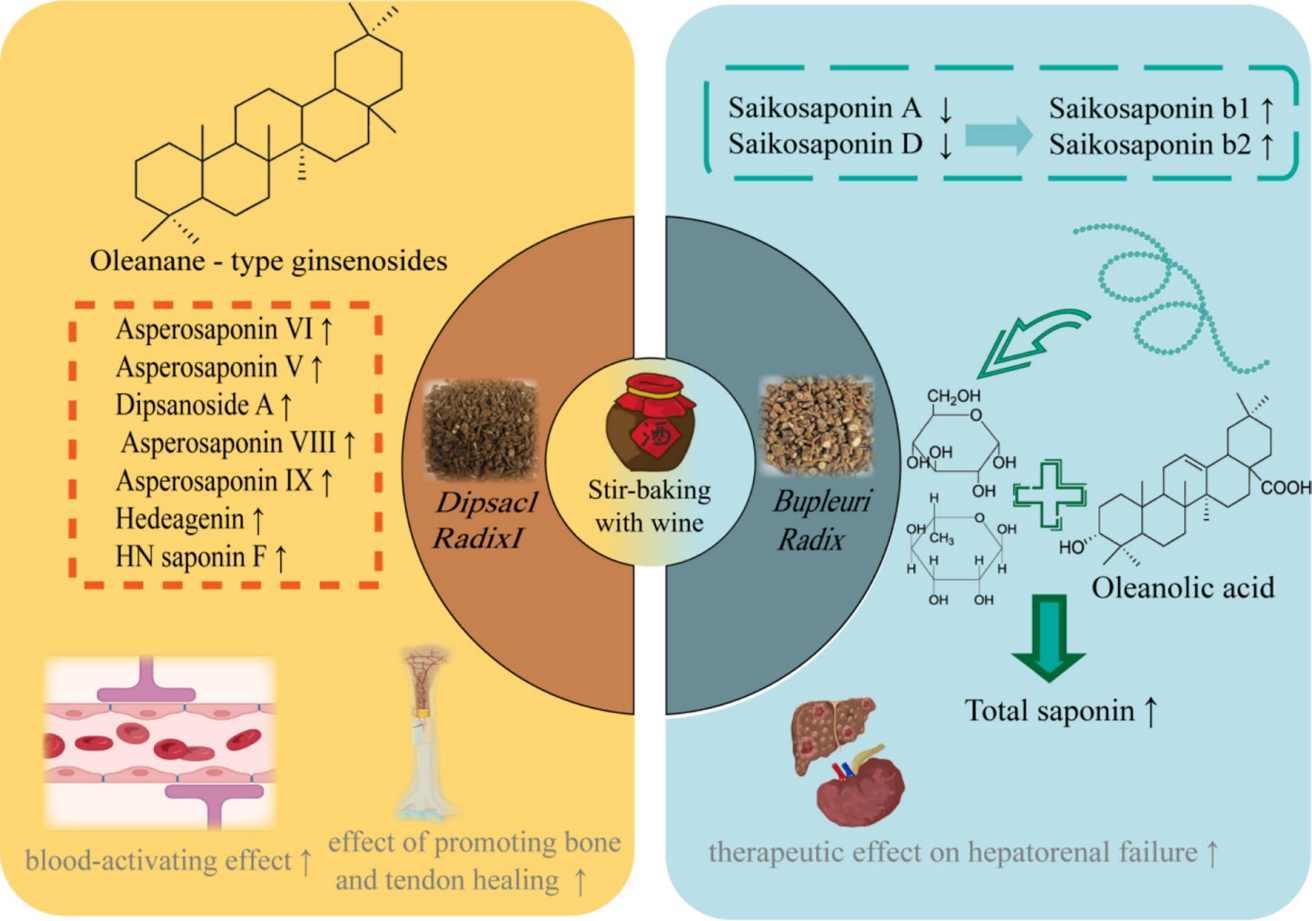
Effects of stir-baking with wine on saponin-rich TCMs

Stir-baking with yellow wine refers to the methods of processing TCMs with wine as an excipient. Yellow wine is hot, sweet and pungent, and fragrant, which can conduct drugs to distributing upwards and has dispersing effect and has the effect of activating blood to dredge collaterals, dispelling wind and cold, guiding the drug’s ascending tendency, and removing the bad odour and modifying the drug’s taste. For many TCMs rich in saponins, stir-baking with wine not only induces changes in saponin components but also enhances their effects of activating blood circulation and collaterals, as well as tonifying the liver and kidneys—*Dipsaci Radix* and *Achyranihis Bidentatae Radix* serve as classic examples (Fig. 3).

*Dipsaci Radix*, which exhibits effects of tonifying the liver and kidneys as well as strengthening bones and muscles, has major active components of triterpenoid saponins [43] that have been demonstrated to treat fractures and osteoporosis [44, 45]. After stir-baking with wine, the contents of asperosaponin VI [46, 47], asperosaponin V, dipsacoside A [48], asperosaponin VIII, asperosaponin IX, hederagenin, HNsaponin F [49], dipsacoside B, and three acetylated analogs of asperosaponin VI increase significantly, thereby enhancing its effects of activating blood circulation, promoting bone and tendon regeneration, and stopping metrorrhagia [50]. *Achyranthes bidentata* exerts effects of tonifying the liver and kidneys, as well as removing blood stasis and dredging meridians [51, 52]. The total saponins of *Achyranthes bidentata* have been widely studied and confirmed to possess relevant pharmacological activities. Most saponins in *Achyranthes bidentata* are oleanolic acid-type triterpenoid saponins, which are structurally similar. During stir-baking with wine, high-temperature heating may induce interconversion among saponin components; concurrently, it causes the degradation of polysaccharides in *Achyranthes bidentata*, and the decomposed monosaccharides such as glucose and rhamnose further combine with the oleanolic acid present in *Achyranthes bidentata* to form triterpenoid saponins, thereby resulting in a decrease in the total polysaccharide content, an increase in the contents of total saponins and ginsenoside Ro in wine-processed *Achyranthes bidentata*, as well as an enhancement of its therapeutic effect on hepatorenal failure [53, 54].

This figure focuses on the stir-baking with wine technique, with *Dipsaci Radix* and *Bupleuri Radix *as examples for analysis. After wine processing, *Dipsaci Radix* promotes saponin dissolution and enhances osteogenic efficacy; *Bupleuri Radix* promotes saponin transformation, increasing the total saponin content and strengthening its effect in treating liver and kidney failure. Furthermore, it reveals the regulatory role of yellow rice wine’s “solubility enhancement and absorption promotion” properties on saponin components and pharmacological effects.

##### Stir-baking with vinegar

**Fig.4:**
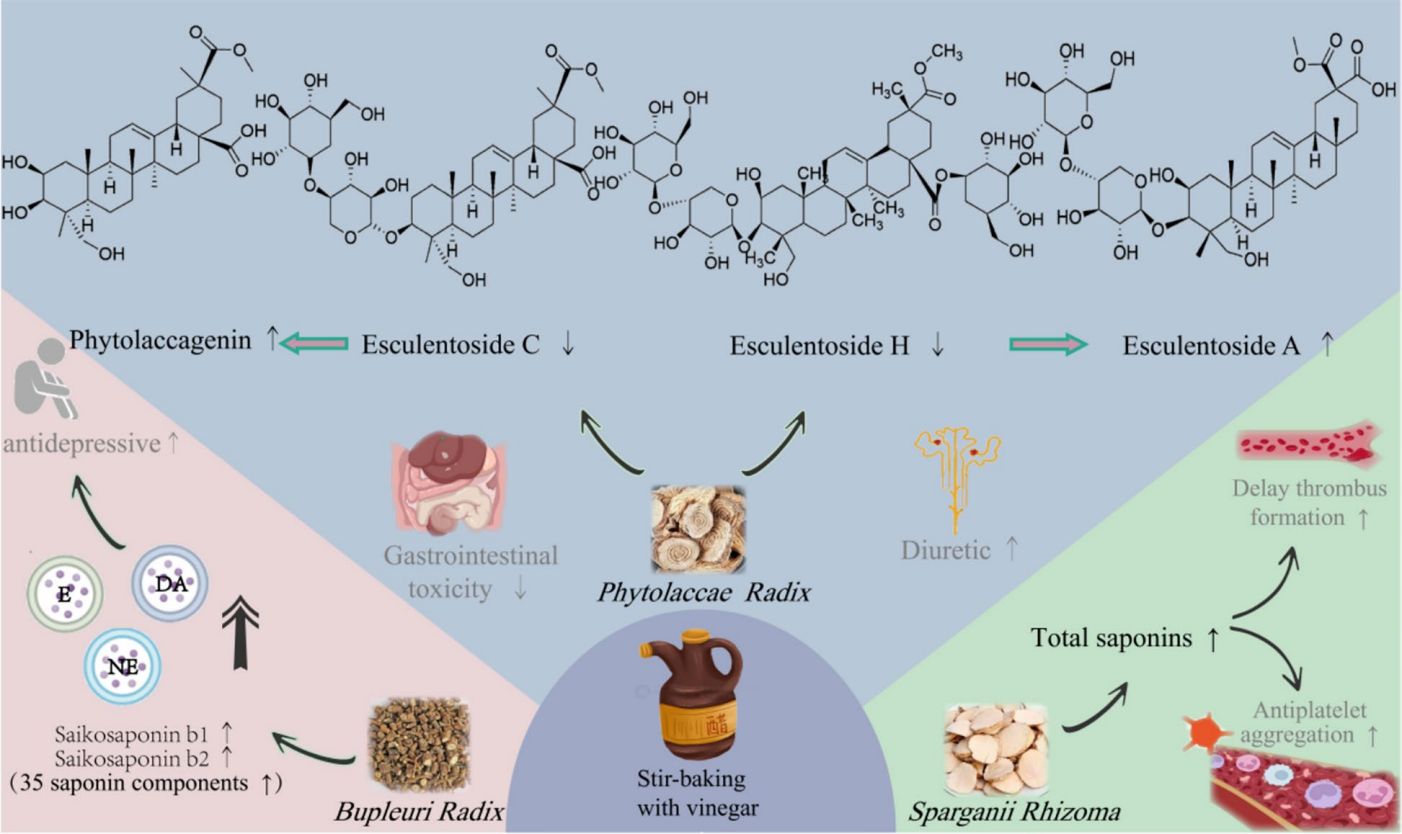
Effects of stir-baking with vinegar on saponin-rich TCMs

Stir-baking with vinegar refers to the method of processing TCMs using vinegar as a processing excipient. Vinegar, which is both edible and medicinal, is warm in nature, sour and bitter in taste, and guides drugs to the liver meridian and blood system, performing functions such as regulating qi, stopping bleeding, promoting water circulation, reducing swelling, removing toxins, dissipating blood stasis to relieve pain, and eliminating bad odors while modifying the drug’s taste, containing organic acids, aldehydes, esters, alcohols, and phenols [55]. Traditionally, vinegar has been widely used in the preparation of drugs for soothing the liver to relieve depression, dissipating blood stasis to alleviate pain, and expelling retained fluid through purgation (Fig. 4).

*Radix Bupleuri*, is a representative herb processed with vinegar which exhibits effects of relieving exterior syndrome to reduce fever, soothing the liver to relieve depression, elevating yang qi, and suppressing malaria. Triterpenoid saponins, the major active components of *Radix Bupleuri*, demonstrate a wide range of biological and pharmacological activities, including analgesic, immunomodulatory, hepatoprotective, anti-inflammatory, antitumor, and antiviral properties [56]. After being processed with vinegar, *Radix Bupleuri* shows an increase in the content of 35 saponin components, among which 21 components (mainly including antidepressant components such as saikosaponin b1 and saikosaponin b2) have more than doubled in content. The changes in saponin components during processing thereby enhance the ability of vinegar-processed *Radix Bupleuri* to regulate emotional factors, leading to increased levels of circulating estrogen [57] as well as dopamine and norepinephrine [58] in the brain of animals, and strengthen its regulatory effect on responses in rats with liver depression models [59]. Another herb whose efficacy is enhanced by vinegar processing is *Sparganii Rhizoma*, which has the effects of breaking blood stasis, promoting qi circulation, resolving food stagnation, and relieving pain; its traditional processing method is vinegar processing, and its total saponins exhibit significant effects such as reducing platelet aggregation and delaying thrombosis, with the content of total saponins increasing after vinegar processing, thereby enhancing its medicinal efficacy [60]. Vinegar processing not only potentiates the therapeutic efficacy but also reduces toxicity. For instance, *Phytolaccae Radix*, a toxic Chinese medicinal herb, is commonly processed with vinegar to detoxify. During vinegar processing, chemical transformations occur under acidic conditions in *Phytolaccae Radix*, leading to a decrease in the contents of toxic components esculentoside B, esculentoside C, and esculentoside H—among which esculentoside H undergoes ester bond hydrolysis under acetic acid conditions and thus generates esculentoside A, the major diuretic component—while the content of esculentoside A increases [61], thus achieving both toxicity reduction and efficacy enhancement.

This figure systematically presents the regulatory effects of the stir-baking with vinegar technique. For *Bupleuri Radix*, it promotes saponin dissolution, enhances the ability to regulate emotional factors, and strengthens antidepressant efficacy. For *Sparganii Rhizoma*, it increases the total saponin content and enhances antithrombotic effects. For *Phytolaccae Radix*, it reduces toxic saponins through acid-catalyzed reactions while retaining diuretic activity. It comprehensively demonstrates the effects of vinegar processing on inducing saponin transformation, enhancing efficacy, and reducing toxicity.

##### Stir-baking with salt water

**Fig.5:**
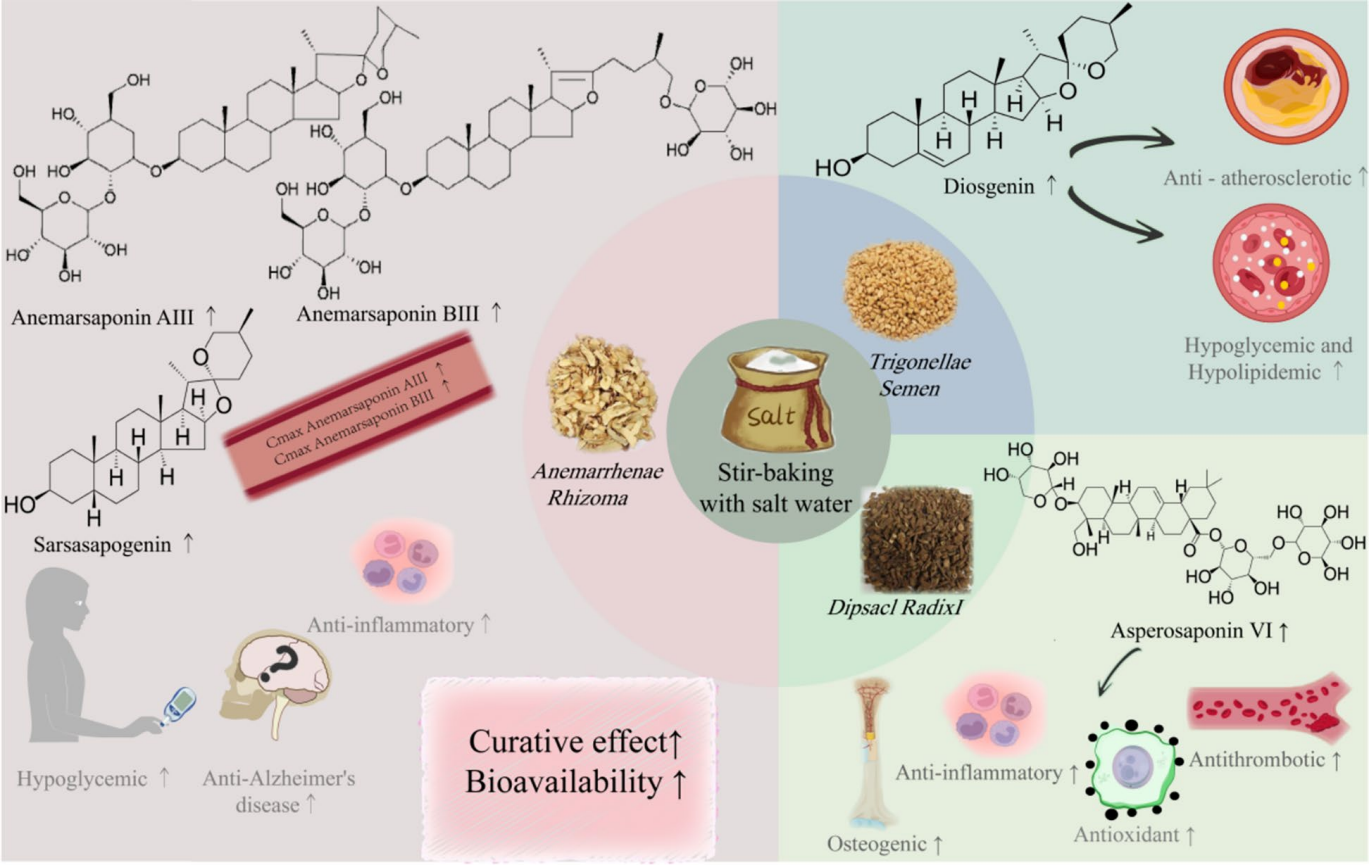
Effects of stir-baking with salt water on saponin-rich TCMs

Stir-baking with salt water refers to the method of processing TCMs using salt water as a processing excipient. Salt is salty in flavor and cold in nature, exhibiting effects of clearing away heat and cooling the blood, softening hard masses to resolve stagnation, moistening dryness to promote defecation, strengthening bones and muscles, guiding herbs to the kidney, as well as preserving and correcting taste. After salt processing, the effects of tonifying the liver and kidneys, nourishing yin to reduce fire, treating hernia to relieve pain, and promoting diuresis can be enhanced (Fig. 5).

*Anemarrhena asphodeloides*, a representative herb processed with salt, exhibits the effects of clearing heat and purging fire, as well as nourishing yin and moistening dryness. Its main active components are steroidal saponins. Smilagenin shows anti-tumor and anti-diabetic activities [62], timosaponin AⅢ and timosaponin BⅢ exert significant hypoglycemic effects [63, 64]. Salt-processing increases the contents of smilagenin [65, 66], timosaponin AⅢ [67], and BⅢ [64–68] and pharmacokinetic studies reveal enhanced absorption of timosaponin AⅢ and BⅢ [69], leading to strengthened effects of *Anemarrhena asphodeloides* in hypoglycemic activity [70], treatment of diabetic cognitive impairment [71], anti-inflammation [72], nourishing yin to clear heat [73], and moistening intestines for laxative purposes [74]. Similarly, there are also TCMs such as *Dipsaci Radix* and *Trigonellae Semen*. After salt-processing, the content of asperosaponin Ⅵ—an active ingredient with osteogenic [75, 76], anti-inflammatory [77], antithrombotic [78], and antioxidant [79] effects in *Dipsaci Radix*—increases significantly [80]. After salt-processing, the content of diosgenin—a bioactive component with hypoglycemic, lipid-lowering [81, 82], and anti-atherosclerotic [83] effects in *Trigonellae Semen*—also increases [84]. All the above results indicate that salt-processing may enhance medicinal efficacy by promoting the dissolution of active substances and improving their bioavailability.

Using *Anemarrhenae Rhizoma*, *Dipsaci Radix*, and *Trigonellae Semen* as research objects, this figure clarifies the processing effects of stir-baking with salt water. For *Anemarrhenae Rhizoma*, it improves saponin dissolution and bioavailability, enhancing pharmacological effects such as hypoglycemia and anti-Alzheimer’s disease. For *Dipsaci Radix*, it promotes the dissolution of osteogenic saponins and strengthens osteogenic and other pharmacological effects. For *Trigonellae Semen*, it increases the content of diosgenin, thereby enhancing lipid-lowering activity. This figure clarifies the effects of salt processing on saponin components, bioavailability, and pharmacological efficacy.

##### Stir-baking with ginger juice

**Fig.6:**
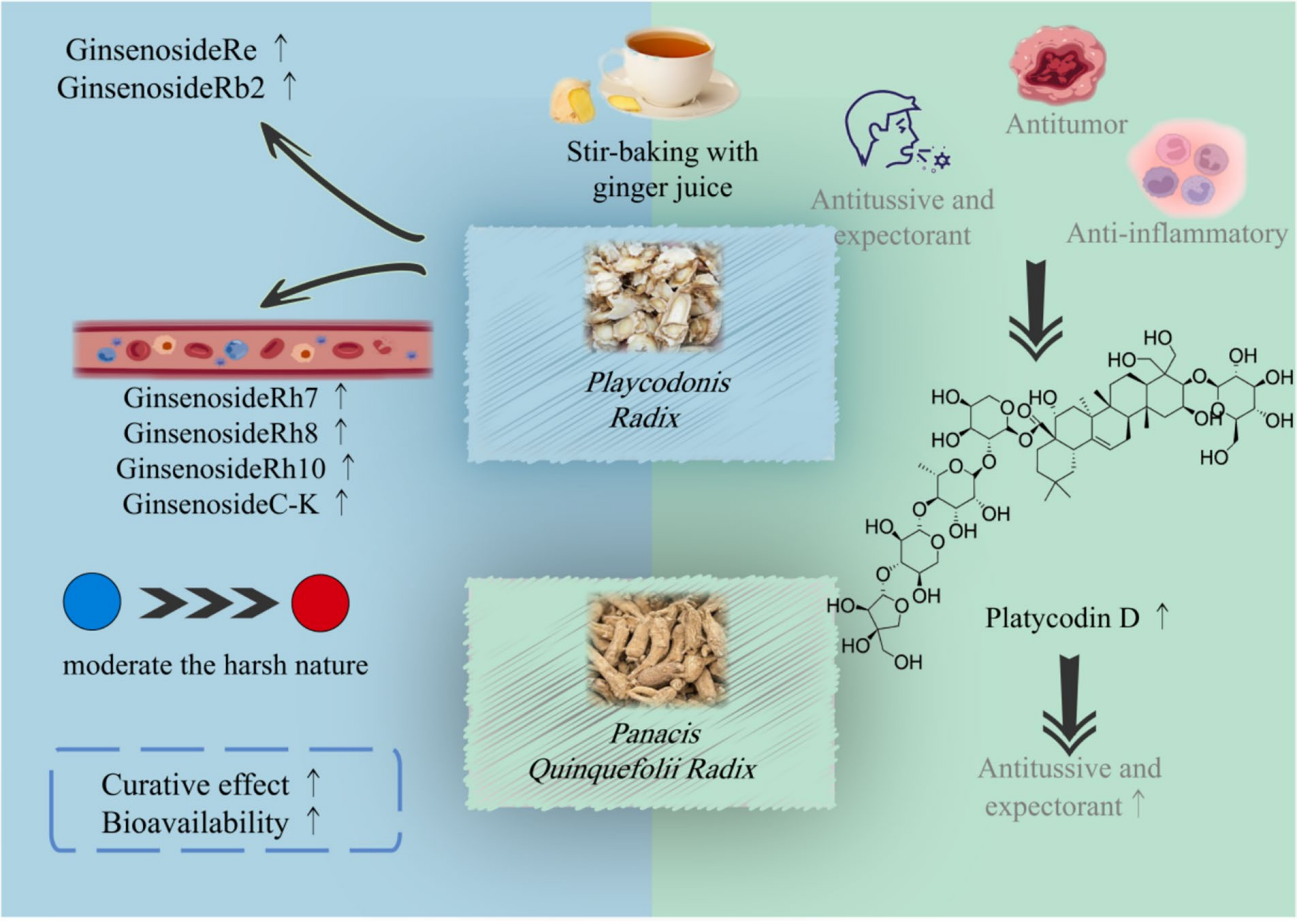
Effects of stir-baking with ginger juice on saponin-rich TCMs

Ginger processing is a general term for the methods of processing TCMs using ginger juice a processing excipient. As early as in the Han Dynasty, ginger juice processing was already in use. Ginger is pungent in flavor and warm in nature, which can warm middle energizer to arrest vomiting and clear up the phlegm to stop coughing. Stir-frying with ginger can reduce or eliminate the toxic and side effects of drugs and correct the biases of drugs such as excessive coldness and strong purgation, as exemplified by *Panax quinquefolius*. It can also enhance therapeutic efficacy, as seen in the case of *Platycodon grandifloras *(Fig. 6).

*Panax quinquefolius* (American ginseng) has the effects of invigorating and tonifying the body, nourishing yin and moistening the lung, and nourishing the stomach to promote fluid production. However, its cold nature restricts its clinical application to a certain extent, while ginger juice, being warm in nature, can moderate this cold bias of *Panax quinquefolius *[85]. The active components in *Panax quinquefolius* are ginsenoside compounds, which exhibit various significant pharmacological effects. After ginger processing, the contents of 28 ginsenoside compounds including Ginsenoside Re and Ginsenoside Rb2 increased significantly. Further analysis of blood-absorbed components revealed that rare ginsenosides such as Ginsenoside Rh7, Ginsenoside Rh8, Ginsenoside Rh10, and Ginsenoside C-K were identified as blood-absorbed components only in the ginger-processed *Panax quinquefolius* group [86]. It is speculated that the ginsenoside components in ginger-processed *Panax quinquefolius* are partially converted into rare ginsenosides in vivo after being absorbed into the blood, thereby exerting better in vivo pharmacological effects. Another herb whose efficacy is enhanced after ginger processing is *Platycodon grandiflorus*. The active component platycodin D in *Platycodon grandiflorus* exerts expectorant, antitussive [87], anti-tumor [88, 89], anti-inflammatory [90, 91], antioxidant [92, 93], and immunomodulatory effects [94]. After ginger processing, the content of platycodin D is higher than that in the raw herb and other processed products [80], resulting in enhanced antitussive and expectorant effects [95].

Taking *Panacis Quinquefolii Radix* and *Platycodonis Radix *as examples, this figure reveals the multiple effects of stir-baking with ginger juice. For *Panacis Quinquefolii Radix*, ginger juice can moderate its cold nature and promote the absorption of rare saponins. For *Platycodonis Radix*, ginger juice increases the content of platycodin D and enhances expectorant and antitussive effects. It clearly demonstrates the effects of ginger processing on moderating the nature of TCMs, promoting saponin transformation, and enhancing efficacy.

##### Stir-baking with honey

**Fig.7:**
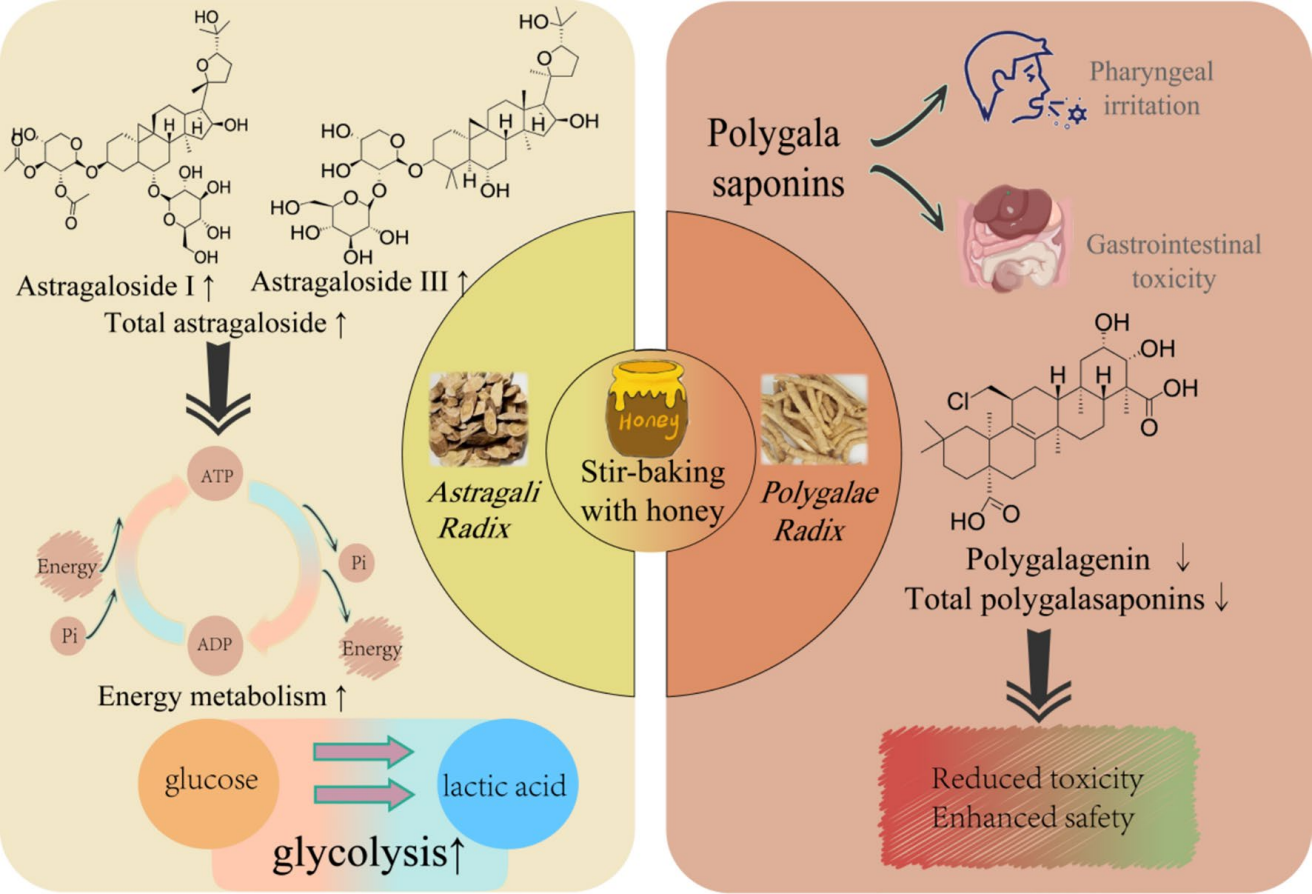
Effects of stir-baking with honey on saponin-rich TCMs

Stir-baking with honey refers to a general term for the methods of processing TCMs using honey as an excipient. Honey, with its sweet taste and neutral nature, exerts effects of nourishing the spleen, moistening the lung to relieve cough, and modify the drugs’ taste. Stir-baking with honey is commonly applied to TCMs for relieving cough and asthma, as well as tonifying the spleen and replenishing qi. In many saponin-rich TCMs, honey processing not only alters saponin components but also enhances the effects of lung-moistening cough-relief and middle-jiao qi-tonification, such as *Astragali Radix*, while reducing toxicity, such as *Polygalae Radix* (Fig. 7).

The effective parts of honey-processed *Astragali Radix* (a representative honey-processed drug) are mainly composed of polysaccharides, saponins, and flavonoids [96]. Saponin components in *Astragali Radix* exhibit functions such as promoting development, enhancing immunity [97], regulating blood glucose, antioxidation, anticancer activity, and neuroprotection [98]. After honey-processing, the contents of astragaloside I, astragaloside III [99], and total astragaloside [100] in *Astragali Radix* increase, enhancing the effects of promoting energy metabolism, glycolysis [101], improving fatigue, and reversing immune dysfunction in rats with spleen qi deficiency induced by dietary restriction [102]. *Polygalae Radix* has a numb taste and causes strong irritation to the throat, along with certain gastrointestinal toxicity [103]. Therefore, it is often used after processing in clinical applications. The polygala saponins in *Polygalae Radix* are the main components causing toxic reactions [104–106], which stimulate gastrointestinal mucosa, damage gastrointestinal tissues [107], induce reflexive vomiting [108], and easily trigger inflammatory cell infiltration and vascular dilation [16]. Tenuigenin inhibits gastrointestinal motility, reduces pepsin activity, and causes digestive system toxic reactions such as abdominal distension [109]. Compared with raw *Polygalae Radix*, honey-processing reduces the contents of total saponins [110] and tenuigenin [106–112], decreases toxicity, and significantly improves safety.

With *Astragali Radix* and *Polygalae Radix *as research objects, this figure illustrates the regulatory value of stir-baking with honey (using honey as an excipient and leveraging its natural ionic liquids and deep eutectic solvents). For *Astragali Radix*, it achieves “efficacy enhancement”; for *Polygalae Radix*, it achieves “toxicity reduction”. It clarifies the differential effects of honey processing on saponin-rich TCMs.

##### Stir-baking with oil

Oil processing, a method of processing TCMs with edible oils (divided into vegetable oils and animal fats) as excipients, can enhance the effects of TCMs in tonifying the kidney and supporting yang. Notoginseng (*Panax notoginsen*g), which possesses the effects of dissipating blood stasis, stopping bleeding, reducing swelling, and relieving pain with saponins as its main active components, can be processed into prepared notoginseng via oil-processing, resulting in increased contents of saponins such as ginsenosides Rg6 and Rg4 [113], which exert certain effects on liver diseases, inflammatory conditions, and cardiovascular disorders [114–116].

### Steaming

**Fig.8:**
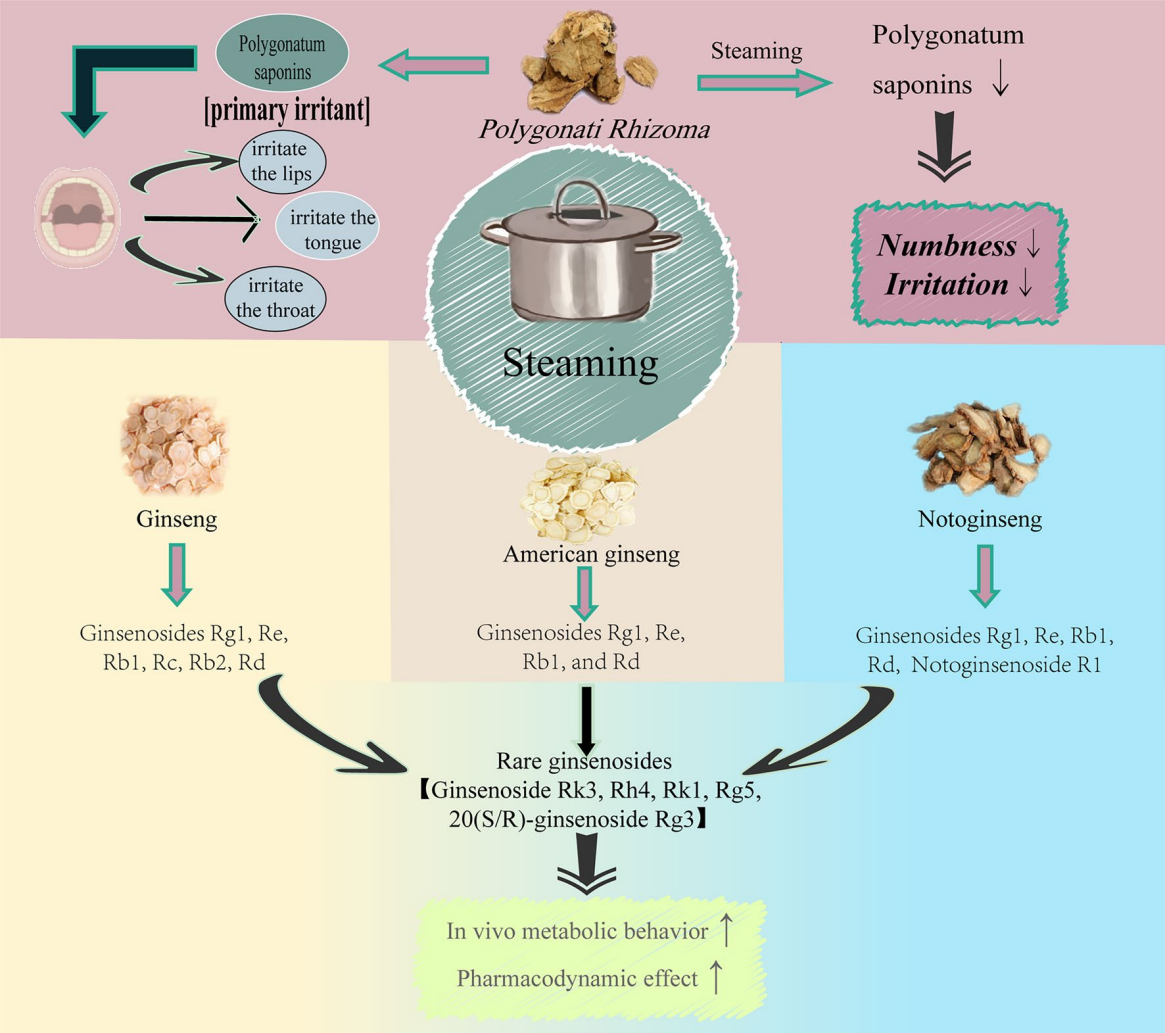
Effects of steaming on saponin-rich TCMs

Steaming is a method of heating TCMs with water vapor, which is often divided into steaming with processing excipients and steaming without processing excipients. Steaming is mainly used for tonic TCMs, as it can strengthen the tonic effects of these medicinal materials—for example, Ginseng, Notoginseng, and American ginseng; it is also applied to toxic TCMs, with steaming reducing the toxicity and side effects of such drugs, such as *Polygonati Rhizoma *(Fig. 8).

Ginseng, American ginseng, and Notoginseng are representative medicinal materials processed by steaming. Ginsenosides Rg1, Re, Rb1, Rc, Rb2, and Rd are the main components of Ginseng; ginsenosides Rg1, Re, Rb1, and Rd are the main components of American ginseng; ginsenosides Rg1, Re, Rb1, Rd, and notoginsenoside R1 are the main components of Notoginseng. After high-temperature steaming, all the main components of these three TCMs undergo hydrolysis, dehydration, and isomerization reactions and thus are converted into rare ginsenosides such as ginsenoside Rk3, Rh4, Rk1, Rg5, and 20(S/R)-ginsenoside Rg3 [117]; for details of the process, sect 3. “Chemical Reactions of Saponins during Processing”. Rare ginsenosides, which are secondary metabolites derived from prototypical ginsenosides, are characterized by greater chemical diversity and abundant biological activities—including anti-cancer [118], anti-inflammatory, anti-cardiovascular disease [119], anti-diabetic, anti-depressant [120], anti-Alzheimer’s disease [121], anti-tumor, anti-aggregatory effects [122], as well as protective effects on the nervous system and liver. Compared with their prototypical counterparts, they exhibit superior in vivo metabolic behavior and pharmacological efficacy [6], making up for the shortcomings of ginsenosides, which generally have poor absorption in the gastrointestinal tract and low bioavailability [123].

After steaming, ginseng shows enhanced anti-aging [124], anti-myocardial hypertrophy [125], and anti-tumor effects [125]; steamed Notoginseng exhibits strengthened antioxidant and hematopoietic activities [126]; and steamed American ginseng demonstrates improved protective effects against myocardial injury [127]. Saponins are widely used in the pharmaceutical and functional food fields due to their significant pharmacological activities; however, it is noteworthy that some saponins can irritate the digestive tract mucosa and exhibit toxicity [128], causing irritation to the lips, tongue, and throat. *Polygonati Rhizoma*, a traditional Chinese medicine, exhibits similar side effects, which may be attributed to the presence of saponins and alkaloids in it—with saponins likely being the main irritants [129]—and there are significant differences in saponin components before and after steaming [130]: nine saponin components present in raw *Polygonati Rhizoma* are not detected in wine-steamed *Polygonati Rhizoma *[131]*.* The reduction in numbness and irritation after steaming may be due to the removal of sugar chains from some highly irritating saponins, or the isomerization of highly irritating saponins into weakly irritating ones.

In summary, steaming achieves the “transformation, efficacy enhancement, and toxicity reduction” of saponin-rich traditional Chinese medicines through the synergistic effect of hydrothermal treatment: it uses water vapor for uniform heating to soften the texture and break down cell walls, thereby promoting the dissolution of saponins; it triggers chemical reactions such as hydrolysis through high temperature and water to realize efficacy enhancement and toxicity reduction; additionally, excipients can be added to directionally regulate the transformation of saponins, providing a suitable material basis for clinical efficacy, making it a core processing method that combines traditional value with modern potential.

Aking *Ginseng Radix et Rhizoma*, *Notoginseng Radix et Rhizoma*, *Panacis Quinquefolii Radix*, and *Polygonati Rhizoma *as examples, this figure systematically explains the core effects of steaming. For *Ginseng Radix et Rhizoma*, *Notoginseng Radix et Rhizoma*, and *Panacis Quinquefolii Radix*, high-temperature steam promotes the hydrolysis, dehydration, and isomerization of saponins, generating highly active rare saponins. For *Polygonati Rhizoma*, it reduces toxicity by removing irritating saponins. This figure comprehensively demonstrates the effects of steaming on saponin transformation, efficacy enhancement, and toxicity reduction in saponin-rich TCMs.

Various processing methods exert influences on saponin-rich Chinese medicinal materials through high temperature and different adjuvants. High temperature can disrupt the cell structure of medicinal materials; after cell fragmentation, the specific surface area increases, thereby accelerating saponin dissolution. Meanwhile, it promotes the chemical structure transformation of saponins, leading to the generation of new active components, an increase in the content of effective ingredients, and a reduction in the content of toxic components. On this basis, different adjuvants further affect saponins by synergizing with high temperature. Yellow wine, via its solvation effect, more readily assists high temperature in triggering saponin reactions. For example, in the 2.3.3.1. “Stir-baking with wine” process applied to *Dipsacus asper* and *Achyranthes bidentata*, it facilitates saponin dissolution and the entry of active components into the bloodstream. The acidic environment of vinegar (containing acetic acid, pH 3.0–4.5) can directionally promote the protonation and cleavage of saponin glycosidic bonds, prioritizing the occurrence of deglycosylation reactions. This is exemplified by the deglycosylation of saikosaponins in *Bupleurum chinense* under vinegar processing conditions, as described in the subsequent section 3.5. “Deglycosylation Reaction”. The sodium ions in salt can alter the osmotic pressure of medicinal material cells, promoting the dissolution of saponin components in the medicine—such as the salt-water stir-baking process (2.3.3.4. “Stir-baking with salt water”) used for *Anemarrhena asphodeloides* and *Dipsacus asper*. Additionally, the weakly alkaline environment of honey (containing fructose, glucose, organic acids, and minerals) exhibits properties similar to natural ionic liquids and deep eutectic solvents, which can enhance saponin solubility. A typical case is the honey-stir-baking process (2.3.3.5. “Stir-baking with honey”) applied to *Astragalus membranaceus* and *Polygala tenuifolia*. Moreover, the components of ginger juice may interact with saponins to improve their solubility. These processing methods optimize saponin-rich Chinese medicinal materials from multiple aspects, embodying the scientific connotation of “enhancing efficacy and reducing toxicity” in traditional Chinese medicinal processing.

## Chemical reactions of saponins during processing

After processing, the saponin components in TCMs undergo changes. Processing provides reaction conditions for saponin transformations, activating the reactive groups and chemical bonds in saponins and thereby triggering corresponding chemical reactions. These reactions follow certain rules and typically include hydrolysis, dehydration, isomerization, cleavage, and desugarization reactions.

### Hydrolysis reaction

Hydrolysis is a type of chemical reaction that refers to the reaction between a compound and water. During this reaction, chemical bonds in saponin molecules undergo cleavage, and the hydrogen ions (H^+^) and hydroxide ions (OH^−^) dissociated from water molecules then combine respectively with the cleaved segments of the compound, generating new components that affect the efficacy of the drug. A common hydrolysis reaction in the processing of TCMs involves the hydrolysis and cleavage of glycosidic or ester bonds in one or more components to form corresponding secondary products. Processing methods such as steaming, boiling, and blanching in TCM processing all involve heat treatment under aqueous conditions, and some require prolonged heating, thus often accompanied by hydrolysis reactions of chemical components. Among these, the most typical examples are the hydrolysis reactions that occur in Ginseng and Notoginseng during the steaming process, and in Licorice during the honey process.

**Fig.9:**
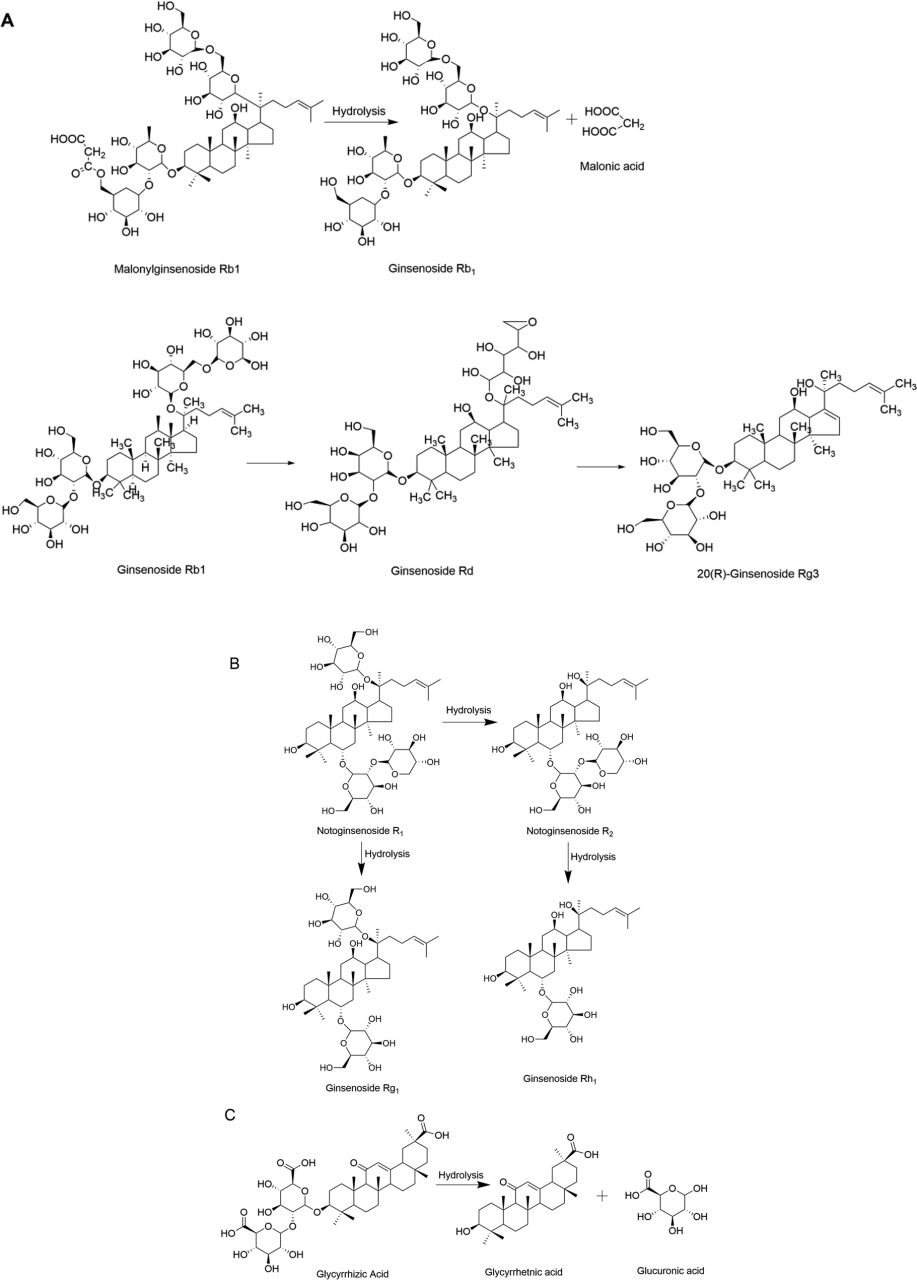
Hydrolysis Reaction. **A** Hydrolysis Reaction of Ginsenoside Rb1; **B** Hydrolysis Reaction of Notoginsenoside R; **C** Hydrolysis Reaction of Glycyrrhizic Acid

During the steaming process of ginseng, saponins are prone to hydrolysis: malonyl ginsenosides Rb1, Rc, Rb2, and Rd undergo hydrolysis upon heating to generate ginsenosides Rb1, Rc, Rb2, Rd, and malonic acid, and with continued heating, the resulting products undergo further hydrolysis to produce more rare ginsenosides [181], thereby enhancing efficacy. Taking ginsenoside Rb1 as an example, the specific reaction mechanism is shown in Fig. 9A. Meanwhile, saponins in many TCMs also undergo hydrolysis reactions during the processing, which enhances their medicinal effects. For example, after steaming of Notoginseng, triterpenoid polysaccharides such as notoginsenoside R and ginsenosides Rg1, Re, Rb1, and Rd often undergo glycosidic bond cleavage and side-chain hydrolysis, generating rare ginsenosides like ginsenoside Rh1 and Rh4 [182]. Taking the hydrolysis reaction of notoginsenoside R as an example, the specific reaction mechanism is shown in Fig. 9B. The above-mentioned changes lead to improvements in blood viscosity, enhancement of immunity, and strengthened qi-tonifying effects of processed Notoginseng. Glycyrrhizic acid, the main component of licorice [183], is a typical triterpenoid saponin with extremely low oral bioavailability of only 4.0%; however, when it undergoes hydrolysis to convert into glycyrrhetinic acid, its bioavailability increases, allowing it to be absorbed into the bloodstream and exert its effects [123]. During the honey-processing of licorice, glycyrrhizic acid is hydrolyzed upon heating to form glycyrrhetinic acid [184], an easily absorbable active component, with the specific reaction mechanism shown in Fig. 9C. Additionally, studies have found that liquid fermentation of licorice using strain HC-12 can also convert glycyrrhizic acid into glycyrrhetinic acid, which enhances anti-inflammatory and analgesic activities and exerts its effects rapidly [185].

### Dehydration reaction

In the processing of TCMs, dehydration reaction refers to a process in which components in medicinal materials lose water molecules and undergo structural changes under specific processing conditions, forming new components. This reaction is usually accompanied by the transformation of functional groups or induces ring system reconstruction, thereby affecting the polarity, activity, or toxicity of pharmacologically active components.

**Fig.10:**
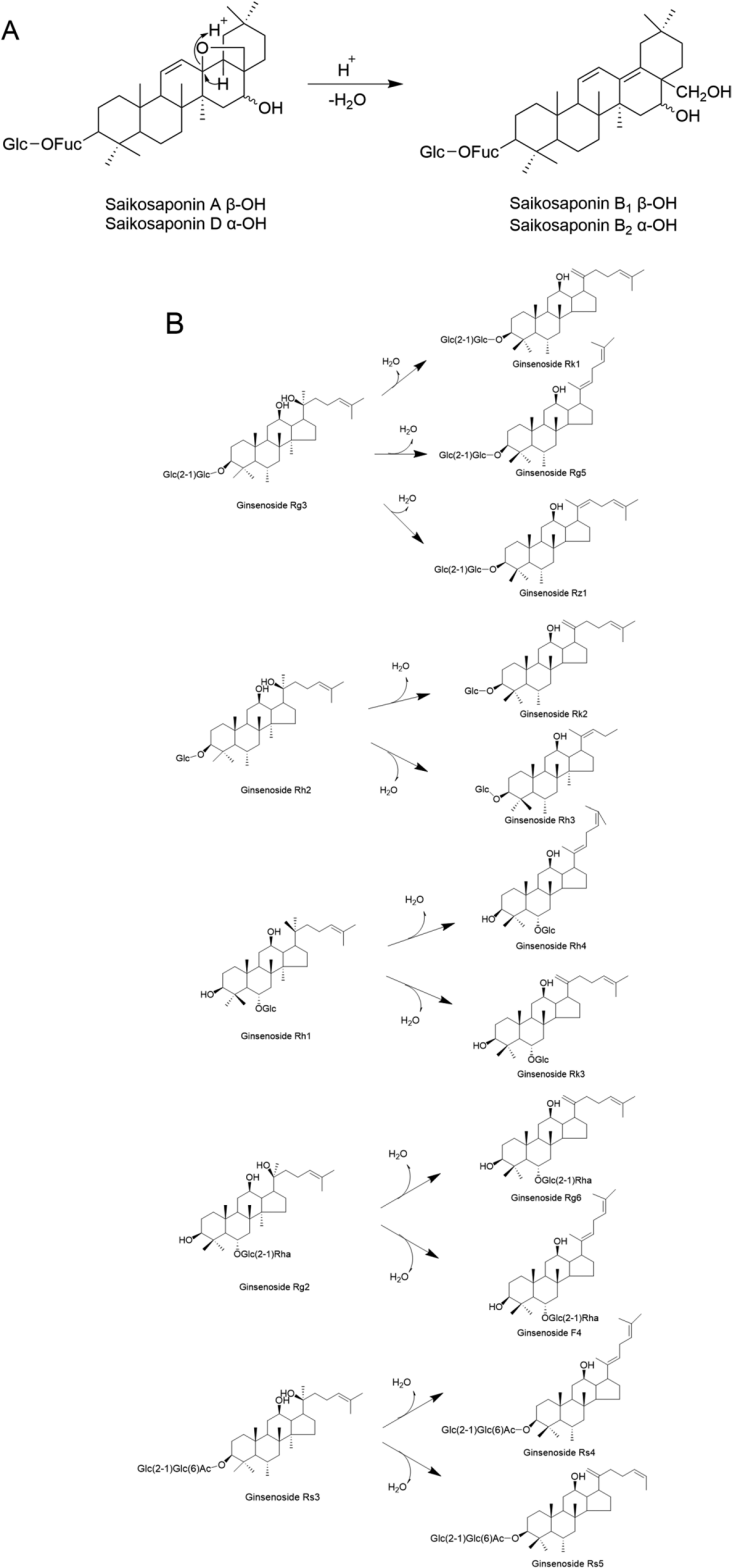
Dehydration Reaction. **A** Dehydration Reaction of Saikosaponins A and D; **B** Dehydration Reaction of Ginsenosides

During the vinegar-processing of *Radix Bupleuri*, the ether rings in the structures of saikosaponins A and D undergo cleavage, followed by dehydration to form saikosaponins b1 and b2 with an isocyclic diene structure [186]. This is a typical dehydration reaction, whose basic process is shown in Fig. 10A. Some saikosaponins are converted from primary to secondary forms, enhancing the effects of soothing the liver and protecting the liver [187].

Dehydration reactions are also all observed in American Ginseng, Ginseng, and Notoginseng during the steaming process, the dehydration of ginsenosides often occurs at C-20, forming double bonds between C-20 and C-21 or C-20 and C-22, leading to positional and geometric isomerism: Ginsenoside Rg3 undergoes dehydration to generate ginsenosides Rk1, Rg5 [188, 189], and Rz1; ginsenoside Rh2 undergoes dehydration to generate ginsenosides Rk2 and Rh3; ginsenoside Rh1 undergoes dehydration to generate ginsenosides Rh4 and Rk3 [177]; ginsenoside Rg2 undergoes dehydration to generate ginsenosides Rg6 and F4 [190]; ginsenoside Rs3 undergoes dehydration to generate ginsenosides Rs4 and Rs5 [191]. The specific reaction mechanism is shown in Fig. 10B.

### Isomerization reaction

Isomerization refers to a reaction in which a chemical substance changes its composition and structure under specific conditions to form a new substance, with the product usually being an isomer of the reactant. Since the bond energies of many isomers differ slightly, they can be converted into each other at room temperature. The isomerization reaction in TCMs processing refers to the simple structural changes that occur in the components of TCMs under processing conditions during the processing process.

**Fig.11:**
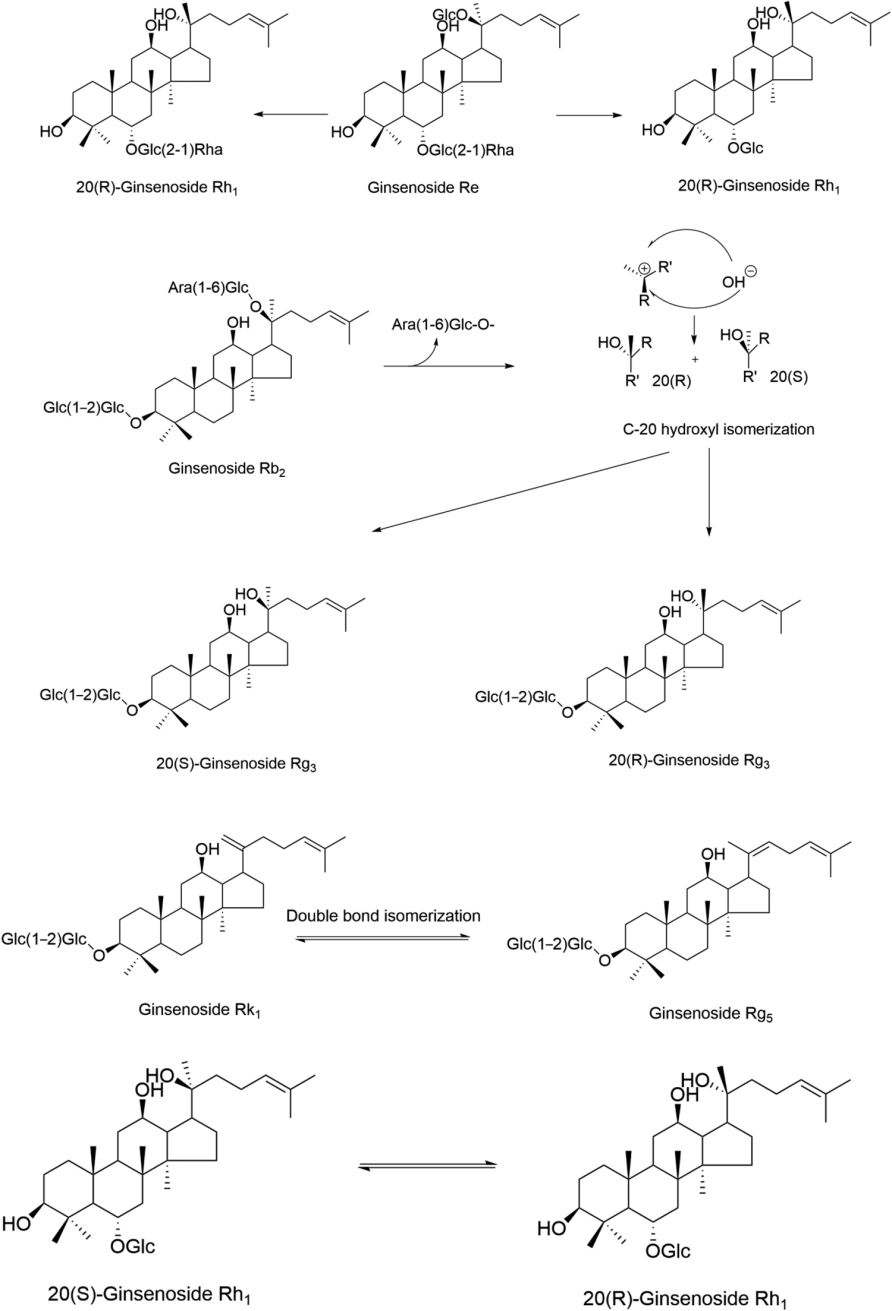
Isomerization Reaction of Ginsenosides

Due to the difference in the spatial arrangement of the C-20 hydroxyl group in ginsenosides, some ginsenosides exist as two isomers (S and R); during processing, the C-20 side chain undergoes cyclization, with the S configuration converting to the R configuration [192], so when ginseng is processed into red ginseng by heating and steaming, some natural S-configured ginsenosides undergo isomerization while being hydrolyzed, transforming into R-configured secondary glycosides. This reaction is the key factor behind the “enhanced pharmacological effect” of Ginseng during steaming—the formation of 20(R)-Rg3 enhances the anti-tumor activity of Ginseng, highlighting the regulatory role of isomerization reactions in saponin activity. For example, during processing, ginsenoside Re loses the glucose at the C-20 position and the rhamnose at the C-3 position to form ginsenoside Rh1; meanwhile, the C-20 position undergoes a configuration change from the original S-configuration to the R-configuration, ultimately generating 20R-ginsenoside Rh1. If only the glucose at the C-20 position is hydrolyzed, with the C-20 position converting from the S-configuration to the R-configuration, 20R-ginsenoside Rg2 is formed. Simultaneously, during steaming, ginsenoside Rb2 can be converted into 20(S)-ginsenoside Rg3 and 20(R)-ginsenoside Rg3, while ginsenoside Rg5 and ginsenoside Rk1 also undergo transformations [173]; similarly, the isomerization of ginsenoside Rh1 occurs during the steaming and baking of ginseng [193]. These reactions collectively represent typical isomerization processes in the processing of TCMs, with the underlying mechanisms illustrated in Fig. 11.

### Cleavage reaction

Cleavage refers to a chemical reaction where a compound undergoes cleavage of its chemical bonds under specific conditions, decomposing into two or more compounds. During the processing of TCMs, some components are prone to cleavage reactions upon heating, with bond cleavage often occurring at chemical bonds with low bond energy and poor stability [194], and the C–O bond is one of the chemical bonds that are relatively susceptible to cleavage.

**Fig.12:**
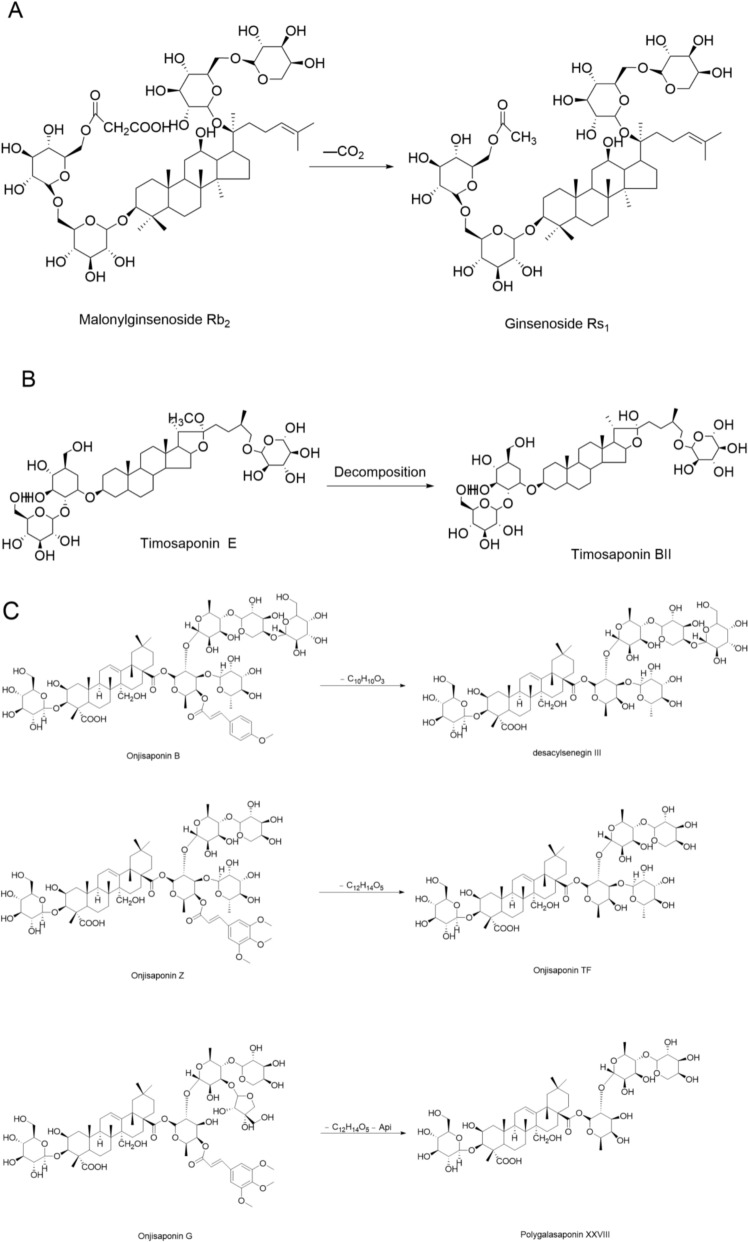
Cleavage Reaction. **A** Cleavage Reaction Cleavage Reaction of Ginsenoside Rb2; **B** Cleavage Reaction of Anemarsaponin E; **C** Cleavage of Polygala Saponins

Fresh ginseng contains ginsenosides such as malonyl-ginsenoside Re, Rb1, Rb2, Rc, and Rd, which undergo decarboxylation cleavage during processing to generate acetyl compounds. For instance, malonyl-ginsenoside Rb2 is converted into acetyl-ginsenoside Rb2 [193], namely ginsenoside Rs1; malonyl-ginsenoside Rc is transformed into acetyl-ginsenoside Rc, namely ginsenoside Rs2. Analysis of their decomposition products reveals the release of carbon dioxide, indicating that this reaction involves the decarboxylation cleavage of the malonic acid moiety in malonyl ginsenosides upon heating. Taking ginsenoside Rb2 as an example, the specific reaction mechanism is shown in Fig. 12A. Cleavage reactions occur during salt-processing of *Anemarrhenae Rhizoma*: timosaponin E undergoes thermal cleavage with methyl group loss to form timosaponin BII [70] (mechanism in Fig. 12B). During boiling of *Polygalae Radix*, Onjisaponins B, Z, and G undergo cleavage to yield desacylsenegin III, Onjisaponin TF, and Polygalasaponin XXVIII, respectively, with their specific reaction mechanisms shown in Fig. 12C.

### Deglycosylation reaction

Saponins are compounds formed by the linkage of aglycones and sugars via glycosidic bonds. The deglycosylation reaction of saponins mainly involves the cleavage of glycosidic bonds. Specifically, deglycosylation reaction refers to a specific reaction that occurs under the conditions of enzymolysis, acidolysis, or pyrolysis (often accompanied by the participation of water), where the glycosidic bonds (C-O bonds) in saponin molecules undergo hydrolytic cleavage, resulting in the detachment of sugar chains and the formation of secondary saponins or aglycones. It is a specific form of hydrolysis reaction. Given that deglycosylation reaction exerts a significant and universal impact on the bioavailability and activity of saponin components such as ginsenosides, it is listed as an independent category separately.

**Fig.13:**
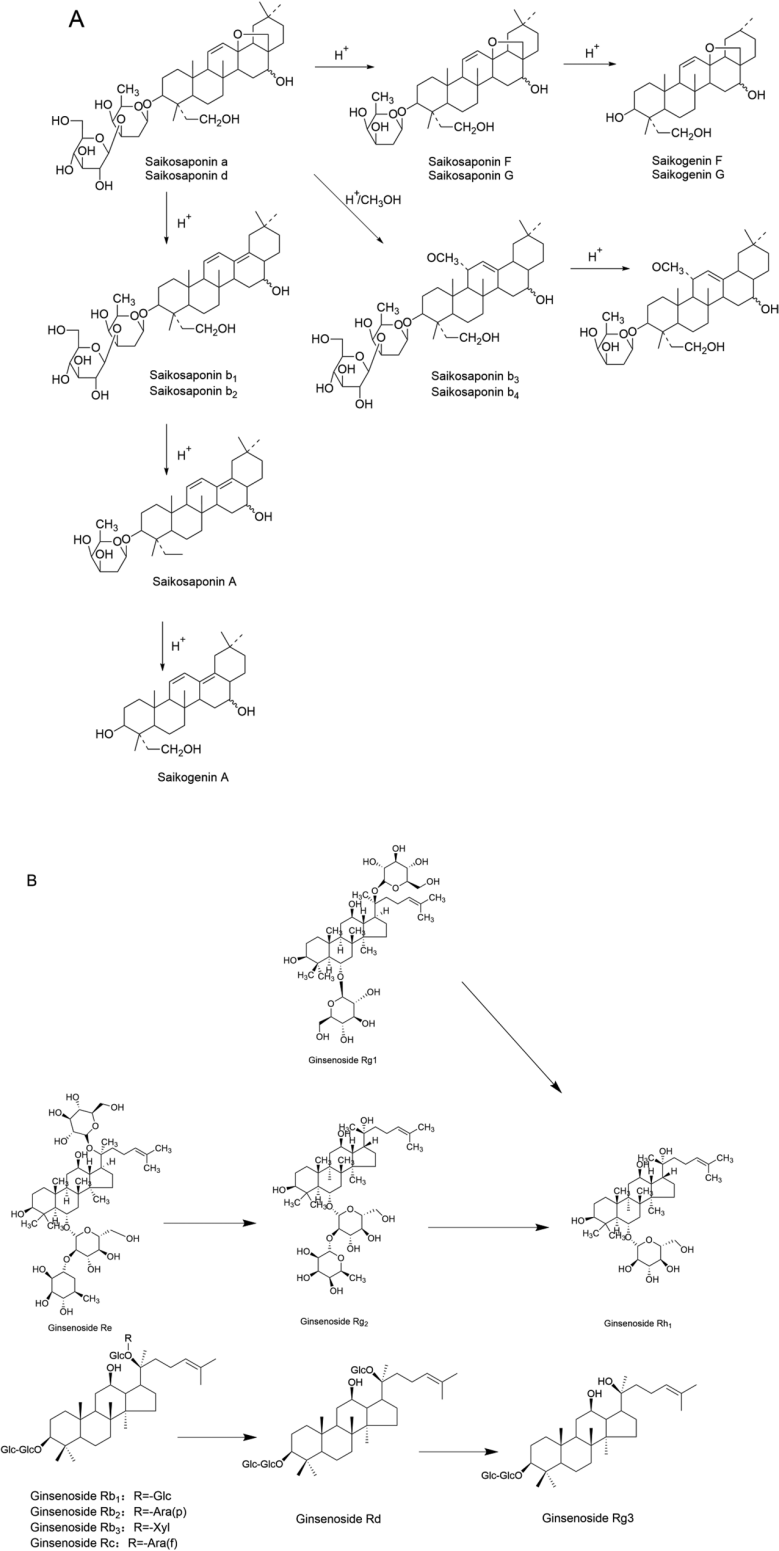

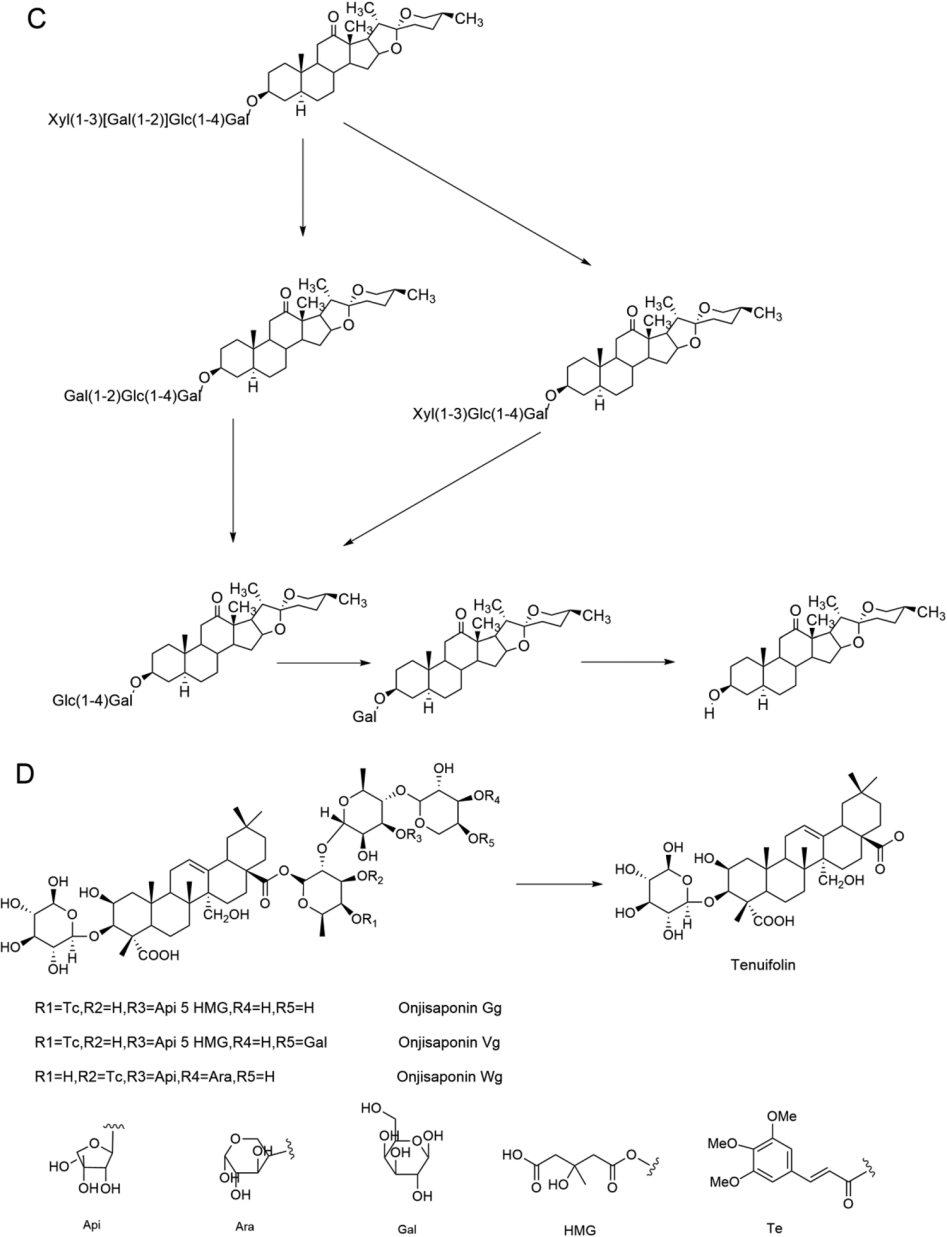
Deglycosylation Reaction. **A** Deglycosylation reaction of saikosaponins; **B** Deglycosylation reaction of ginsenosides; **C** Deglycosylation reaction of tribulosides; **D** Deglycosylation reaction of polygala saponins

This reaction plays a core role in both the vinegar-processing of *Bupleuri Radix* and the stir-baking of *Tribulus terrestris*. During the vinegar-processing of *Bupleuri Radix*, under acidic and heated conditions, the glycosidic atoms of saikosaponins undergo protonation, causing the cleavage of glycosidic bonds, a reduction or complete removal of glycosyl groups, and the formation of secondary saponins or aglycones—which enhances the liver-soothing and depression-relieving activity and verifies the mechanism by which acidic environments accelerate the cleavage of glycosidic bonds—with the specific reaction mechanism illustrated in Fig. 13A. Ginsenosides with high contents in ginseng include Rb1, Rc, Rb2, Rd, Re, and Rg1 [195]; however, their oral bioavailability is relatively low [196]. The presence of glycosyl groups is the main factor contributing to differences in absorption among various ginsenosides [197], as ginsenosides with multiple glycosides are often difficult to absorb in vivo, and the main forms of ginsenosides that are absorbed into the bloodstream and exhibit medicinal effects are rare saponins (Rg3, Rg5, Rh2, Rh3, Rk1, Rk2) and glycosides produced after deglycosylation [198, 199]. During the steaming process of ginseng, ginsenosides with more glycosyl groups undergo deglycosylation reaction, resulting in the removal of glycosyl groups and the formation of ginsenosides with fewer glycosyl groups; specifically, during steaming, ginsenoside Rd can undergo deglycosylation at the C20 position to generate ginsenoside Rg3, and furthermore, ginsenoside Rg3 can undergo further glycosyl release at the C3 position, thereby generating ginsenoside Rh2 [200–202]. During stir-baking ginseng with rice, numerous deglycosylation reactions also occur: ginsenoside Re loses the C-20 glycosyl group to convert into the rare ginsenoside 20(S)-Rg2; ginsenoside Rg1 loses the C-20 glycosyl group to transform into the rare ginsenosides 20(S)-Rh1 and 20(R)-Rh1; ginsenosides Rb1, Rb2, Rb3, and Rc lose the glycosyl group at the C-20 or C-3 position to convert into the rare ginsenosides 20(S)-Rg3, 20(R)-Rg3, or F2 [36, 193]. Similar reactions occur during the steaming process [203, 204], with the specific reaction mechanisms illustrated in Fig. 13B. Similar to ginseng, *Tribulus terrestris* undergoes deglycosylation during stir-baking: tribuloside D loses sugar moieties to generate hecogenin, where the deglycosylation preferentially occurs at the terminal sugar of the sugar chain, followed by sequential removal of multiple glycosyl groups from the outside to the inside of the chain, ultimately forming the aglycone [29]; the specific reaction mechanism is illustrated in Fig. 13C. Under conditions of adding water and heating, the toxic saponin components contained in *Polygalae Radix* undergo deglycosylation to convert into tenuifolin with lower toxicity, and the specific reaction mechanism is shown in Fig. 13D. Additionally, studies have found that the characteristic saponins in *Polygonati Rhizoma* may undergo deglycosylation during wine-steaming.

## Conclusions and prospects

The processing technology of TCMs, which has been continuously improved over more than 2000 years, can enhance the efficacy of TCMs and reduce their toxicity. To summarize, various processing methods exert unique effects on saponin-rich TCMs. Among them, cleaning can affect saponin-rich TCMs by removing impurities and non-medicinal parts; appropriate softening, cutting, and drying methods can influence such TCMs by promoting the dissolution of saponin components and reducing their loss. Simple stir-baking can act on saponin-rich TCMs through high temperature, while stir-baking with liquid or solid excipients can exert effects via high temperature, water, and various excipients; steaming, utilizing high temperature and water, can affect saponin-rich TCMs by accelerating saponin dissolution, simultaneously inducing a series of chemical reactions of saponins to generate new active components, increasing the content of effective components, and reducing the content of toxic components.

Currently, there is a growing body of research both domestically and internationally on the effects of processing on saponin-rich TCMs. However, most existing studies focus on changes in the content and structure of saponins during traditional processing, while there is insufficient research on the pharmacodynamic changes caused by processing-induced saponin alterations, the impact of processing on the pharmacokinetics of saponin components, and the effects of innovative processing technologies on saponin-rich TCMs, all of which require further in-depth investigation:

(1) Impact of Saponin Changes on Pharmacodynamic Alterations during Processing.

The ultimate goal of clarifying the chemical mechanisms during TCM processing is to reveal the material basis underlying the changes in efficacy before and after processing, thereby providing guidance for the rational clinical application of TCM decoction pieces. Currently, research on the effects of processing on saponin-rich TCM mainly focuses on chemical changes; in subsequent studies, it is necessary to closely integrate chemical composition analysis with pharmacodynamic mechanism research. This integration can not only comprehensively reveal the chemical reactions and changes in chemical components occurring during TCM processing but also systematically elucidate the impact of these component changes on the clinical efficacy of TCM decoction pieces.

(2) Impact of Processing on the Pharmacokinetics of Saponin Components.

The efficacy of TCMs is influenced not only by their chemical components but also by the pharmacokinetic processes within the human body. On the one hand, under specific TCM processing conditions, a series of chemical reactions occur to generate components that are easily absorbed; on the other hand, the unique formulation properties of excipients can form specific complexes with active ingredients—for instance, honey, as an excipient in honey-processing, exhibits properties resembling natural deep eutectic solvents (NADES)—and such complexes can regulate the solubility and permeability of active ingredients, thereby improving their in vivo pharmacokinetics. Current research on the effect of processing on the pharmacokinetics of saponins mostly remains at the macroscopic level of “enhanced absorption efficiency”. Further in-depth exploration is required to elaborate on the mechanisms of key links in in vivo metabolism—such as investigating the role of the gut microbiota in the metabolism of saponins after processing, and exploring changes in the activity of the hepatic metabolic enzyme system.

(3) Influence of Innovative Processing Technologies on Saponin-Rich TCMs.

Traditional processing techniques are plagued by issues such as “low conversion efficiency, unstable products, and significant batch-to-batch variations”, so there is an urgent need to integrate modern technological means to achieve a breakthrough in saponin components from “random conversion” to “directional regulation” and promote the modernization and upgrading of processing techniques. Directional processing refers to the design of new processing methods based on the reaction mechanisms underlying chemical component changes and their biological activities during processing, with the purpose of directionally transforming the chemical components of TCMs into highly active, low-toxicity, or even non-toxic components. Directional processing of saponin-rich TCMs based on the unique properties of saponins can maximize the efficacy of TCMs. We can explore specific technical methods to optimize saponin conversion: enzyme-assisted processing, for instance, can screen specific enzyme preparations and optimize enzymatic hydrolysis processes to meet the key conversion requirement of saponin deglycosylation for rare saponin production; microwave-ultrasound synergistic processing can enhance the efficiency of saponin structural conversion; and microbial fermentation processing can construct a “microbial community-saponin-pharmacodynamic effect” directional regulation system, which utilizes the metabolic activity of microorganisms to achieve precise conversion of saponins and enhancement of pharmacodynamic effects.

Conclusion Driven by both inheriting the essence and promoting the innovative development of TCM, research on TCM processing has become increasingly in-depth. As an important chemical component in TCMs, saponins play an indispensable role in the application of TCMs. This paper systematically reviews the effects of various processing methods—including cleaning, cutting, simple stir-baking, stir-baking with solid excipients, stir-baking with liquid excipients (Stir-baking with yellow wine, Stir-baking with vinegar, Stir-baking with salt water, Stir-baking with ginger juice, Stir-baking with honey, Stir-baking with oil), and steaming—on saponin-rich TCMs, as well as the series of chemical reactions that saponins undergo during processing, such as hydrolysis, dehydration, cleavage, isomerization, and deglycosylation. This review not only systematically clarifies the intrinsic relationship between processing methods and changes in saponin components but also provides a reference for further exploring the processing mechanisms of saponin-rich TCMs, optimizing processing techniques to maximize efficacy, and reducing potential toxicity.

## Data Availability

Not applicable.

## Abbreviation

TCM: Traditional Chinese Medicine
